# Comparative mitogenomic analyses of *Amazona* parrots and Psittaciformes

**DOI:** 10.1590/1678-4685-GMB-2017-0023

**Published:** 2018

**Authors:** Nicholas Costa Barroso Lima, André Elias Rodrigues Soares, Luiz Gonzaga de Paula Almeida, Igor Rodrigues da Costa, Fernanda Midori Sato, Patricia Schneider, Alexandre Aleixo, Maria Paula Schneider, Fabrício R. Santos, Claudio V. Mello, Cristina Miyaki, Ana Tereza R. Vasconcelos, Francisco Prosdocimi

**Affiliations:** ^1^ Laboratório de Genômica e Biodiversidade, Instituto de Bioquímica Médica Leopoldo de Meis, Universidade Federal do Rio de Janeiro, Rio de Janeiro, RJ, Brazil; ^2^ Laboratório de Bioinformática, Laboratório Nacional de Computação Científica, Petrópolis, RJ, Brazil; ^3^ Departamento de Genética, Centro de Ciências Biológicas, Universidade Federal do Pará, Belém, PA, Brazil; ^4^ Laboratório de Genética e Evolução Molecular de Aves, Departamento de Genética e Biologia Evolutiva, Instituto de Biociências, Universidade de São Paulo, SP, Brazil; ^5^ Coordenação de Zoologia, Museu Paraense Emilio Goeldi, Belém, PA, Brazil; ^6^ Departamento de Biologia Geral, Instituto de Ciências Biológicas, Universidade Federal de Minas Gerais, Belo Horizonte, MG, Brazil; ^7^ Department of Behavioral Neuroscience, Oregon Health & Science University, Portland, OR, USA; ^8^ Departamento de Bioquímica e Biologia Molecular, Universidade Federal do Ceará, Fortaleza, CE, Brazil

**Keywords:** Next-generation-sequencing, Psittacidae, mitogenomics, control region

## Abstract

Amazon parrots are long-lived birds with highly developed cognitive skills, including vocal learning. Several parrot mitogenomes have been sequenced, but important aspects of their organization and evolution are not fully understood or have limited experimental support. The main aim of the present study was to describe the mitogenome of the blue-fronted Amazon, *Amazona aestiva*, and compare it to other mitogenomes from the genus *Amazona* and the order Psittaciformes. We observed that mitogenomes are highly conserved among Amazon parrots, and a detailed analysis of their duplicated control regions revealed conserved blocks. Population level analyses indicated that the specimen analyzed here seems to be close to *A. aestiva* individuals from Bahia state. Evolutionary relationships of 41 Psittaciformes species and three outgroups were inferred by BEAST. All relationships were retrieved with high support.

## Introduction

Vertebrate mitochondrial genomes (mitogenomes) consist of a circular DNA molecule of about 16 Kb that contains 37 intronless genes. This tightly packed organization with little overlap of genomic features is thought to be the result of selective pressure (Mindell, 1999; Boore, 1999; Ingman and Gyllensten, 2009). The chicken mitogenome was the first avian one to be completely sequenced and annotated (Desjardins and Morais, 1990) and revealed a remarkable difference in gene order in comparison to other vertebrate mitogenomes. Its gene arrangement was initially named as “typical” or “ancestral” avian gene order (Desjardins and Morais, 1990). Since then, hundreds of other avian mitochondrial genomes have been described. In October, 2017, 635 complete mitogenomes were available for Aves in GenBank.

Psittaciformes is one of the most conspicuous avian orders, with species characterized by high longevity (Munshi-South *et al.*, 2006; Young *et al.*, 2012) and advanced cognitive abilities (Pepperberg, 1990; Pepperberg and Funk, 1990; Borsari and Ottoni, 2005), including vocal learning (Farabaugh, 1996; Brauth *et al.*, 1997; Pepperberg, 2002, 2010; Plummer and Striedter, 2002). Regarding the mitogenome, many clades of the family Psittacidae present duplicate copies of the control region (CR) as a result of at least six independent duplication events (Schirtzinger *et al.*, 2012). The Amazon parrots, genus *Amazona*, are among the psittacids that have duplicated CRs, as initially shown by Eberhard *et al.* (2001). The duplication event that gave rise to a duplicated CR in these parrots also resulted in two pseudogenes, pseudo-ND6 and pseudo-tRNA-Glu at the 5’-end of CR1 (Eberhard and Wright, 2016). Mitogenomes have been fully sequenced for only two *Amazona* species, the yellow-shouldered parrot, *Amazona barbadensis* (Urantowka *et al.*, 2013) and the yellow-crowned amazon, *Amazona ochrocephala* (Eberhard *et al.*, 2001).

In order to thoroughly describe and compare mitogenomes of Amazon parrots, we sequenced, assembled and annotated for the first time the complete mitogenome of the blue-fronted parrot (also known as turquoise-fronted parrot or blue-fronted amazon), *Amazona aestiva*. This effort was conducted in the context of the whole genome sequencing project that is under way for this species. We also compared the two CRs of *A. aestiva* with all CRs available for Amazon parrots and identified conserved domains, sequence motifs, and substitution patterns by comparative genomics approaches using both bioinformatics algorithms and manual annotation. Furthermore, all 40 mitogenomes of Psittaciformes species available (as of October, 2017) were used in phylogenomic analyses. Finally, as previous genetic studies suggest that *A. aestiva* and *A. ochrocephala* are not reciprocally monophyletic (Eberhard *et al.*, 2004; Ribas *et al.*, 2007; Caparroz *et al.*, 2009; Chaves *et al.*, 2014), we compared the sequence of the COI gene from the mitogenome described here with those of other individuals of *A. aestiva* and *A. ochrocephala* from various localities in South America to confirm if this individual has a typical *A. aestiva* sequence.

## Materials and Methods

### Sample collection and DNA sequencing

A blood sample was previously (2013) obtained by venipuncture from a captive born male *Amazona aestiva* according to a procedure approved by the Animal Ethics Committee of the Universidade Federal de Minas Gerais (UFMG, 202/2007). This individual (FVVF132) is still alive and legally owned by a private breeder, and its blood sample (B04212) is deposited at UFMG’s Centro de Coleções Taxonômicas in Brazil. Total DNA was extracted using DNeasy Blood & Tissue Kit (Qiagen). Three libraries with insert sizes of 200 bp, 3 Kbp, and 5 Kbp (Table 1) were sequenced in an Illumina HiSeq for the assembly of the nuclear genome. We mapped all the resulting reads on the mitogenome of *Amazona barbadensis* (GenBank accession number JX524615.1) using Newbler (v 2.9) in order to select putative mitochondrial DNA reads.

**Table 1:** Number of reads per *A. aestiva* sequencing library. Total number of raw reads per library, reads that were mapped to the *Amazona barbadensis* mitogenome, and total number of reads used in the assembly of the mitogenome *A. aestiva*.

Library	Raw reads	Mapped reads	Assembled reads
200 bp	398,656,732	25,638	15,194
3 Kbp	160,000,000	3,310	844
5 Kbp	417,221,300	13,320	2,967
Total	975,878,032	42,268	19,005

### Assembly and annotation of the blue-fronted parrot mitochondrial genome

Reads that mapped against the *A. barbadensis* mitogenome were selected and assembled using MASURCA (v 2.3.2) ([Bibr B58] Zimin *et al.*, 2013) to produce a circular mitogenome. A total of 19,005 reads were assembled (Table 1) and sequence annotation was performed using MITOS Web Server (Bernt *et al.*, 2013). Artemis Genome Browser (Rutherford *et al.*, 2000) was used for manual annotation of mitogenomic features. We ran tRNAscan-SE (Lowe and Eddy, 1997) to confirm tRNA annotation. BLAST searches of amino acid sequences were performed to check for precise gene boundaries. Tablet (Milne *et al.*, 2013) was used to verify the sequence coverage along the assembled mitogenome. Conserved regions within the control region – CR - (ETAS, boxes and conserved sequence blocks) were defined by manual annotation based on an alignment of the CR from five taxa of the genus *Amazona*.

### Identification of control region domains and conserved boxes

After manual annotation of the CRs from four *Amazona* taxa (*A. aestiva, A. barbadensis, A. farinosa, A. ochrocephala auropalliata* and *A. ochrocephala oratrix*), we defined the precise boundaries of the domains as follows: the limit between domains I and II was defined using a 45 nucleotide window graph (Ruokonen and Kvist, 2002; ADN riche en software), where the number of adenines dropped to a minimum and the number of guanines started to increase (Figure S1). We also used the beginning of the F-box, which marks the start of a long stretch of conserved sequence in the alignment, for determining this boundary. To define the limit between domains II and III we followed Ruokonen and Kvist (2002) and considered the start of block CSB-1 as the boundary. Therefore, domain II was placed upstream of CSB-1 and domain III was defined as downstream of its first base. The identification of CSBs and conserved boxes was performed by manual inspection of alignments of these elements to the two copies of the CR in *A. aestiva* and other species, in agreement with previous definitions (Eberhard and Wright, 2016).

### Whole mitogenome alignment of Amazon parrots

The *A. aestiva* mitogenome was aligned to the mitogenomes of *A. barbadensis* and *A. ochrocephala* (GenBank accession number NC_027840.1). NUCmer was used for the alignment, and delta-filter and show-snps, from the same program package (Kurtz *et al.*, 2004), were used to mark nucleotide differences in the pairwise comparisons between the mitogenomes of *A. aestiva* and the other two species. The location and type of mismatches were tabulated and verified relative to the genomic features of the *A. aestiva* mitogenome (Table 2).

**Table 2:** Number of single nucleotide polymorphisms when comparing the mitogenome of *A. aestiva* against the mitogenomes of *A. barbadensis* and *A. ochrocephala*.

Mitogenome feature	*A. aestiva* /	*A. aestiva* /
	*A. barbadensis*	*A. ochrocephala*
CDS^*^	272	92
rRNA	10	3
tRNA	32	9
Control Region	203	130
Transitions	405	158
Transversions	27	9
Indel^*^	91	69
Synonymous	226	74
Missense	46	18
Nonsense	0	0
Codon Position 1	49	14
Codon Position 2	16	8
Codon Position 3	207	70

* CDS = coding sequence; Indel = nucleotide insertion or deletion

### Psittaciformes mitochondrial phylogenomics

We aligned all the complete mitochondrial genomes from 40 species of the order Psittaciformes from GenBank (October, 2017; Table S1), our *A. aestiva* mitochondrion plus three avian outgroups (chicken, zebra finch and peregrine falcon) using MUSCLE (Edgar, 2004), and visually inspected the alignment in SeaView v4.5.4 (Gouy *et al.*, 2010). We partitioned the alignment in order to accommodate for the variable evolutionary rates along the different regions of the mitochondrion. For our phylogenetic analyses we used all coding genes as well as the 12S and 16S ribosomal RNA genes, which accounts for the majority of the mitochondrial genomes. We split the dataset into four partitions: 12S and 16S ribosomal genes in one partition, and three partitions for the concatenated coding genes, according to their codon position. We used PartitionFinder (Lanfear *et al.*, 2017) to select the evolutionary model for each partition. We estimated the Psittaciformes phylogenetic relationships using BEAST v1.8.4(Drummond *et al.*, 2012). We assumed a lognormal uncorrelated relaxed clock (Drummond *et al.*, 2006) for each partition, and a GTR+G (4 categories) nucleotide substitution model, with a Birth-Death speciation process (Gernhard, 2008) for the tree prior. We calibrated the molecular clock by placing three distinct normal priors on the age of divergence between: 1) Neoaves and Galloanseres, 87 Mya ± 10 Mya; 2) Falconiformes and Passerimorpha, 60 Mya ± 5 Mya; and 3) Psittaciformes and Passeriformes, 50 Mya ± 5 Mya (Jarvis *et al.*, 2014; Prum *et al.*, 2015). We ran three distinct MCMC chains for 50 million states, discarding the first 10% as burn-in. We inspected for convergence using Tracer v1.6, and built a maximum clade credibility tree using TreeAnnotator v1.8 (Drummond *et al.*, 2012).

### Haplotype network

Previous molecular phylogenies have shown that *A. aestiva* and *A. ochrocephala* are not reciprocally monophyletic (Eberhard *et al.*, 2004; Ribas *et al.*, 2007; Caparroz *et al.*, 2009; Chaves *et al.*, 2014). Also, the parents of the specimen whose mitogenome is being described here were apprehended from the illegal trade and their origin is unknown. Therefore, in order to identify the possible origin of this specimen, we added its partial cytochrome oxidase I (COI) sequence into a matrix of 108 sequences of 506 bp from individuals of the *A. aestiva*/*A. ochrocephala* complex with known geographic origin and available in GenBank (Eberhard *et al.*, 2004; Ribas *et al.*, 2007; Caparroz *et al.*, 2009) (Table S3). The alignment was performed using ClustalW in MEGA6. The haplotype network was constructed by median joining using Network v 4.6 (Bandelt *et al.*, 1999). We also used the webpage DNA Surveillance for species identification of Brazilian parrots (Baker *et al.*, 2003; Chaves *et al.*, 2014) to verify the species of our specimen based on their ND2 sequence. To ratify the other two approaches we aligned the COI gene sequences from 108 *Amazona* specimens (Table S3) using MUSCLE (Edgar, 2004). The resulting alignment contained 622 sites, 29 of which were variable and 17 informative. We estimated the coalescent history of the mitochondrial genomes of the *Amazona aestiva* based on this alignment using BEAST v1.8.4 (Drummond *et al.*, 2012). We used a HKY+G nucleotide substitution model and the skyline plot model coalescent process (Drummond *et al.*, 2005). We assumed a strict molecular clock, and calibrated the tree using a lineage-specific evolutionary rate for the *Amazonas sp*. (1.2510-8 substitutions per site per year), as inferred by Nabholz *et al.* (2016). We ran three MCMC chains for 20 million states, with a 10% burn-in. We inspected the results of all chains to observe for convergence in [Bibr B59] [Bibr B60]Tracer v1.6.

We also estimated Tajima’s D neutrality test (Tajima’s D = 1.031), with *p*-value (p = 0.80) calculated using 1000 simulations in Arlequin v3.5 (Excoffier and Lischer, 2010). The *p*-value was not statistically significant, with no evidence for selection, and the observed frequency of rare alleles was similar to the expected value.

## Results

### Read mapping and features of the mitochondrial genome of *Amazona aestiva*

**Figure 1:**
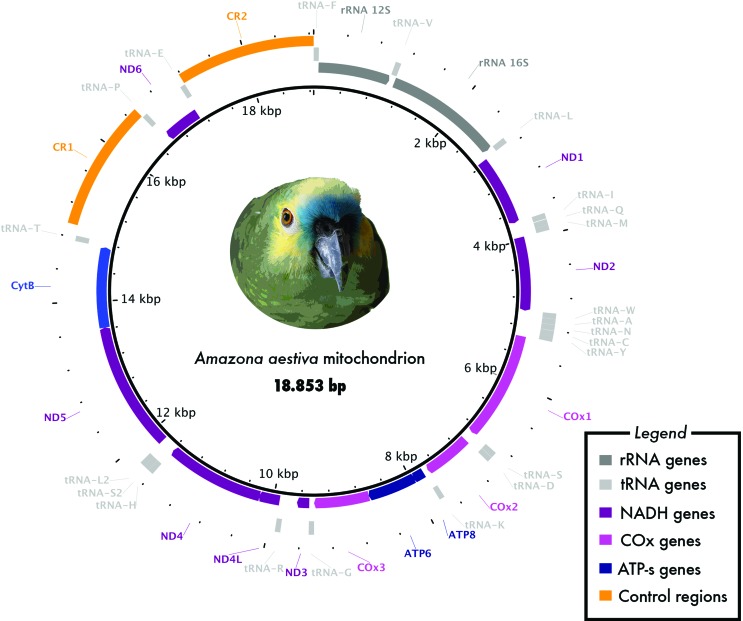
Mitochondrial genome of *Amazona aestiva*. Major genomic features are shown. Colors follow the legend chart.

A total of 42,268 reads of *A. aestiva* mapped onto the mitochondrial genome of *A. barbadensis* (Table 1). The assembled mitogenome was obtained from 19,005 reads, with an average coverage of 183-fold and a maximum coverage of 302-fold. The resulting *A. aestiva* mitogenome consisted of a circular molecule of 18,853 bp (GenBank accession number NC_033336, Figure 1). The Light-strand (L-strand) presented 5,692 adenines, 5,986 cytosines, 2,681 guanines and 4,494 thymines. We found 41 mitogenomic features, including protein coding genes, pseudogenes, tRNAs, rRNAs and two control regions (Table S2).

We found that the mitochondrial L-strand, defined as the A+C-rich strand (Munn, 1975; Taanman, 1999; Vinograd *et al.*, 1963), encodes the majority of the genes in the *A. aestiva* mitogenome. We annotated in the L-strand 14 tRNAs, 2 rRNA, 12 protein coding genes, 2 pseudogenes and 2 control regions (CRs). The Heavy-strand (H-strand) contained the remaining 8 tRNAs and 1 protein coding gene, that of NADH dehydrogenase subunit 6. Of the protein coding genes, 4 had stop codons after the polyadenylation site (Table S2, Figure 1), consistent with Ojala *et al.* (1981).

Consistent with the findings of Urantowka *et al.* (2013) and Eberhard and Wright (2016), we found that the mitogenome of *A. aestiva* follows the general *Amazona* mitochondrial gene order, confirming a CR duplication and pseudogenes for tRNA-Glu and ND6. Specifically, the gene order in this region was CYTB/tRNA^Thr^/ND6^pseudo^/ tRNA^Glu-pseudo^/CR1/tRNA^Pro^/ND6/tRNA^Glu^/CR2/tRNA^Phe^/12s-rRNA. The two CRs of *A. aestiva* were shorter than the ones from *A. barbadensis,* and CR1 was shorter than CR2. The species difference in CR sizes is due to the different number of tandem repeats near the 3’-end of the CRs. The pseudogenes for ND6 and tRNA-Glu presented few differences in comparison with the *A. barbadensis* ones.

### Description of control region domains and conserved sequence motifs

**Figure 2:**
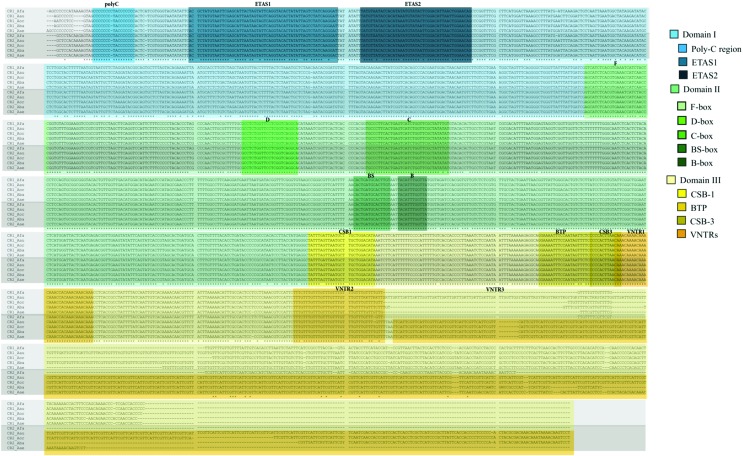
Conserved motifs in the alignment of CRs of *Amazona aestiva* (Aae, KT361659), *A. barbadensis* (Aba, JX524615), *A. farinosa* (Afa, AF338821), *A. ochrocephala oratrix* (Aor, AF338820), and *A. ochrocephala auropalliata* (Aau AF338819).

In vertebrates, CRs are normally divided into three domains (Figure 2). Domain I contains Extended Termination Associated Sequences (ETAS), domain II presents sequence blocks named from A to F, and domain III has conserved sequence blocks (CSB) numbered from 1 to 3 (Ruokonen and Kvist, 2002). In the *A. aestiva* mitogenome we observed that the consensus sequences of sequence boxes and CBSs are quite similar to each other, despite some differences (Table 3). In domain I we identified the poly-C tract (Table 2), with a central TA pair conserved in all CRs but with varying numbers of Cs. We could define ETAS sequences 1 and 2 by alignment of the homologous sequences of *A. aestiva* with those of *A. ochrocephala auropalliata*, obtained from Eberhard and Wright (2016). Domain II had five conserved boxes: F, D, C, Bird similarity box (Bsb) and B. All consensus sequences from these boxes were conserved, with minor changes (Table 2, Figure 2). Domain III presented two out of three CSBs. CSB-1, which was used to define the border between domains II and III, was nearly identical in all *Amazona* species studied, with a shift from an internal AG to GA in the CRs of *A. farinosa*. As observed in other avian CRs, CSB-2 was absent (Desjardins and Morais, 1990). CSB-3 was identical among all CRs compared. We also identified a palindromic sequence that may be the bidirectional transcription promoter as verified by Eberhard and Wright (2016). Domain III also presented three different microsatellite repeats referred to as variable number tandem repeats (VNTRs). VNTR1 presented four CAAA repeats followed by a single internal repeat of CCA and three more CAAA repeats, and was conserved in all *Amazona* CRs compared. VNTR2 presented eight tetranucleotide microsatellite repeats of TTTG that were conserved in all species except in *A. farinosa*, whose first repeat was TTTC. VNTR3 was composed of several repetitions of an 8 nucleotide motif (TTCATTCG) that was absent in all CR1s but present in almost all CR2s, except for that of *A. farinosa*. Most species differences in CRs within the *Amazona* genus were in Domain III (similarity of 76% over 367 sites without gaps; 824 total sites) and were due to the VNTRs, which varied in length. Domain II was highly conserved (similarity of 93.9% over 609 sites without gaps; 610 total sites) and Domain I showed considerable variation (similarity of 78% over 431 sites without gaps; 436 total sites), though not as much as observed in Domain III. Within the same species, the two CR copies were almost identical in Domains I and II, but presented size and repeat differences in Domain III (Table 2 and Figure 2).

**Table 3:** Consensus sequences of conserved motifs in the Control Region (CR) of four *Amazona* species and their respective positions in the mitogenome of *A. aestiva*. Similarity is given as the percentage of identical nucleotides in the consensus of each motif.

	CR1		CR2			
	Start	End	Start	End	Consensus^*^	Similarity
Domain I						
Poly-C	14972	14986	17198	17213	CCCCCCCTACCCCCC(C)	100%
ETAS1	15008	15066	17235	17293	ACKCTATGTAATTCGRRCATTAMTARYAKTCAGGTACAYTATAYYAGYCTATCRRGRRY	73%
ETAS2	15075	15119	17302	17345	YATGTWAYRCCACATADATGTATAMTYGGACATTAAYTGGAACAG	75%
Domain II						
F-box	15404	15429	17631	17656	RKCATCTCACGTGAAAYCATCWACCC	75%
D-box	15507	15529	17734	17755	GCCTCTGGTTCCTCGGTCAGGCA	100%
C-box	15556	15589	17783	17816	TGCCCTTCACTGAGTCATCTGGTTCGCTATWTRT	99,95%
Bird Similarity Box	15791	15805	18018	18032	CACTGATGCACTTGT	100%
B-box	15809	15820	18035	18047	TACATTTGGTTA	100%
Domain III						
CSB-1	16014	16039	18241	18266	TATTTRRTTAATGCTTGCTGGACATA	99,93%
BTP	16105	16126	18332	18353	GGAAAATTTCCAATARTTYTCC	99,01%
CSB-3	16126	16142	18353	18369	CCCCACTTAACAAACAA	100%

* All consensuses are on the 5’-3’ orientation on the L-strand sequence. BTP: Bidirectional Transcription Promoter

### Comparative mitogenomics of Amazon parrots

Alignment of the complete mitogenomes of Amazon parrots (*Amazona aestiva*, *A. barbadensis* and *A. ochrocephala*) revealed greater differences between *A. aestiva* and *A. barbadensis*, with 523 mismatches, including indels. Most of these mismatches were in coding regions. In contrast, *A. aestiva* and *A. ochrocephala* presented 236 mismatches, mostly in the CR.

Comparative analyses of individual loci from the three species revealed that ND5 had the highest number of mismatches, most of them synonymous. It was possible to identify 3 indels at the C-terminal of the *A. barbadensis* ND5. This includes a CT insertion that resulted in a frameshift leading to the substitution of a methionine codon for a leucine codon as well as a premature stop codon. However, since this insertion occurs within the 10 last predicted residues, it is possible that it does not cause major problems in protein folding or function (see also Tables S4, S5 and S6). Species differences in tRNAs and rRNAs were not common but were more frequent between *A. aestiva* and *A. barbadensis*.

### Psittaciformes phylogeny

**Figure 3:**
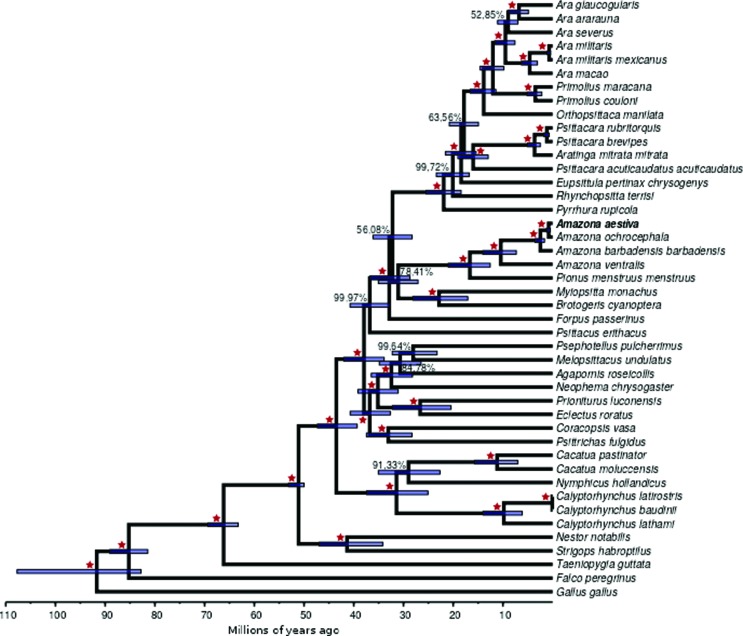
Bayesian tree from all Psittaciformes with mitogenome available and three outgroups. Red stars mark branches with 100% support. Values lower than 100% are shown.

The phylogeny tree obtained (Figure 3) is in accordance with previously published phylogenies of Psittaciformes (Tavares *et al.*, 2006; Schirtzinger *et al.*, 2012). As expected, the outgroups (except for chicken, which was used to root the trees) were placed outside the Psittaciformes ingroup. Furthermore, the sister-taxa relationship between Psittaciformes and Passeriformes was recovered and is congruent with phylogenomic results (Jarvis *et al.*, 2014). Some particular clades were recovered with high support inside the Psittaciformes clade. These were, first, the New Zealand parrots that clustered *Strigops habroptilus* and *Nestor notabilis* in a previously described basal clade of Psittaciformes (Tavares *et al.*, 2006; Wright *et al.*, 2008). A second well-supported clade contained various species of cockatoos from Australasia, consistent with its monophyly. The last higher clade recovered with high support contained all *Amazona* sampled.

The close relationship between the genera *Cacatua* and *Calyptorhynchus,* the genera *Amazona* (Eberhard *et al.*, 2004; Ribas *et al.*, 2007) and *Ara* (Schweizer *et al.*, 2014) were recovered as previously described. Our results were also congruent with the recent taxonomic proposal for the genera *Psittacara*, *Thectocercus*, *Eupsittula* and *Aratinga* (Remsen Jr *et al.*, 2013).

### Analysis of taxonomic status

**Figure 4:**
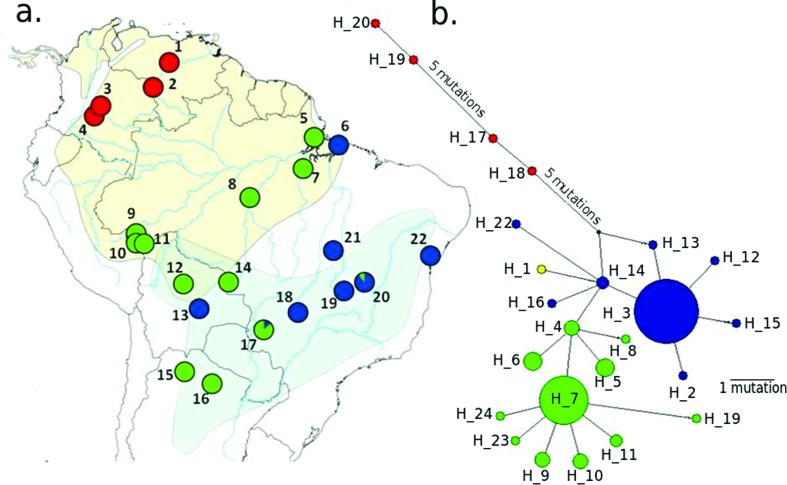
Map and haplotype network. (a) Map showing the distribution of *A. aestiva* (light blue) and *A. ochrocephala* (yellow). Circles and numbers indicate the localities sampled (Table S3). Circle colors represent the frequency of observed lineages on the haplotype network for each locality. (b) Haplotype network with colors representing the three main lineages. Red - *A. ochrocephala*; Green - mixed population of *A. ochrocephala* and *A. aestiva*, with the majority being from the former; Blue - mixed population of *A. aestiva* and *A. ochrocephala*, with the majority being from the former. The COI sequence from the individual whose mitogenome was described (FVVF132) is in yellow.

**Figure 5:**
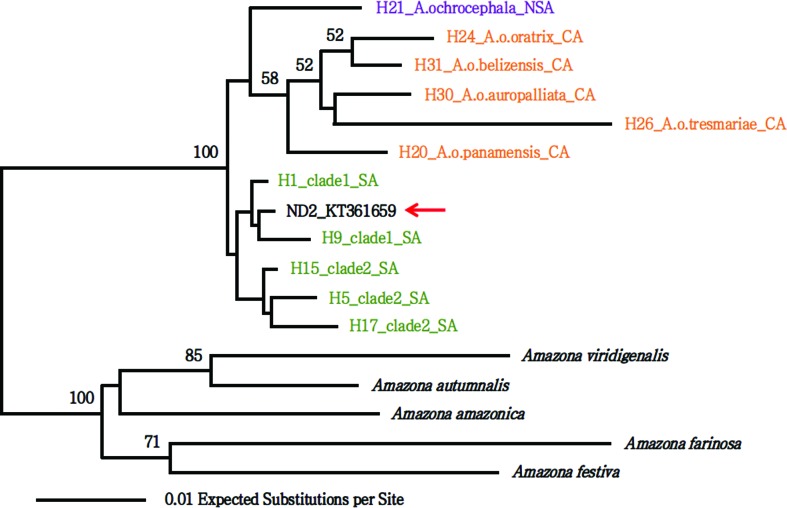
Tree of the DNA Surveillance for species identification of Brazilian parrots. The ID in bold and marked with a red arrow corresponds to the individual analyzed here (FVVF132). Note that the closest related haplotype is H9_clade1_SA.

**Figure 6:**
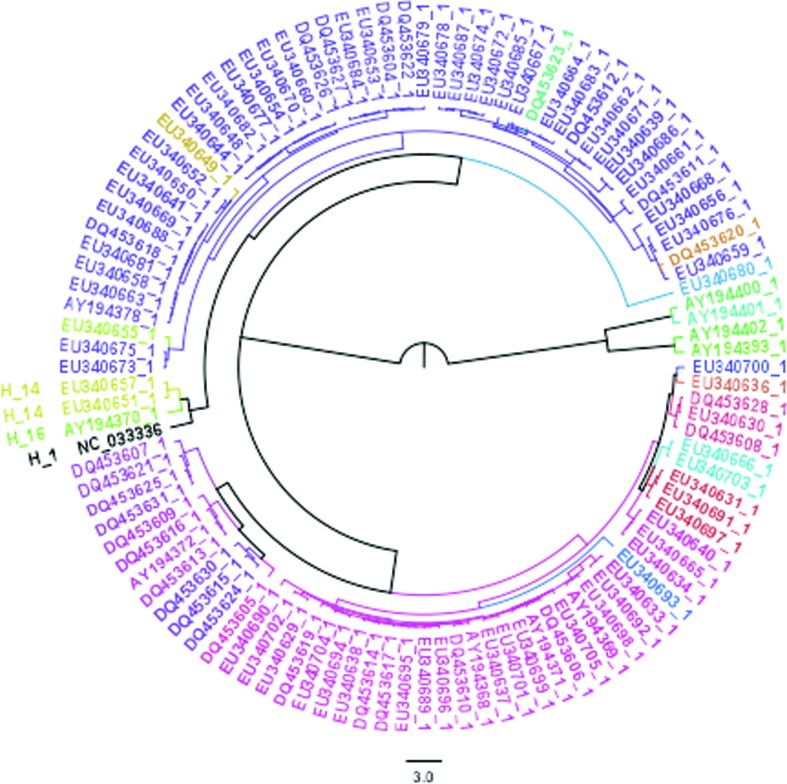
Coalescent-based COI tree. The placement of the COI sequence from the individual described here (H_1, NC_033336) is consistent with our findings using the haplotype network and the DNA Surveillance identification of Brazilian parrots. We also marked the three closest sequences from haplotypes H_14 and H_16.

The specimen sequenced here presents a typical external morphology of *Amazona aestiva aestiva* (Figure S2). The haplotype network based on mitochondrial COI sequences separated this individual (H_1 in Figure 4) by a single mutation from individuals of *A. aestiva* from the Brazilian state of Bahia, haplotype 14 (H_14, Figure 4). Unfortunately, the subspecies of the individuals with H_14 is unknown (no information in GenBank records, nor in associated publications). The DNA Surveillance tree (Figure 5) placed this individual as closely related to the H9_clade1_SA, which presents sequences of captive *A. aestiva* specimens from the Brazilian states Bahia, Tocantins, Minas Gerais, Distrito Federal, and Goiás. The tree built from COI sequences using BEAST (Figure 6) placed this individual in a branch closely related to COIs from haplotype H_14 and H_16, which are also separated from H_14 by one mutation.

## Discussion

We have assembled the complete mitogenome of *A. aestiva* by selecting mitochondrial reads from the whole genome sequencing reads. The majority of the protein coding genes and tRNAs were found to be encoded by the strand that presents the highest amount of A+C, the L-strand (Lima and Prosdocimi, 2018). We recovered the two copies of the CR, which is known to be an AT-rich sequence implicated in the initiation and termination of replication and transcription (Shadel and Clayton, 1997; Gibson *et al.*, 2005). In this region, a truncated H-strand replication product (7S DNA) pairs with its complementary sequence on the L-strand and displaces the H-strand forming a loop, known as displacement-loop or simply D-loop (Nicholls and Minczuk, 2014). Based on the chicken mitogenome (Schirtzinger *et al.*, 2012), the ancestral location of the CR in the avian mitogenome is thought to be between the tRNA-Pro and tRNA-Phe, which corresponds to the Amazon parrot CR2. Thus, the CR conventionally described as CR1 is likely a result of a duplication event that inserted this CR1 and its associated pseudogenes into the mitochondrial genome of an ancestral parrot population that gave rise to the genus *Amazona*. Based on the mitogenome of *A. aestiva*, we suggest that the 1,692 bases between the end of the tRNA-Thr and the start of the tRNA-Pro most likely correspond to the duplicated block. Differences in the length of two CRs in Amazon parrots are due to the presence of a variable number of tandem repeats on the 3’-end (Figure 2).

The two mitochondrial CRs of the genus *Amazona* share an overall 84,5% identity, both within and across species, with high conservation of sequence motifs. This observation is consistent with the possibility that both CR copies may be functional and appear to be evolving in concert, as originally suggested by Eberhard *et al.* (2001). In chicken, the preferred start sites for mitochondrial DNA replication are regions flanking the CR (Reyes *et al.*, 2005). If the same is true for *Amazona* and if both CR copies are functional, it is possible that the mitogenome of this genus may have a higher replication rate when compared to other mitochondria with single CRs. The presence of more efficient mitochondrial DNA replication could in principle increase the expression of components of the electron transport chain, which would concur with the high metabolic rates of parrots (Munshi-South and Wilkinson*,* 2010). Interestingly, preliminary genomic results by Wirthlin *et al.* (2018) point to selective pressure in superoxide dismutase genes, suggesting the importance of antioxidant protection mechanisms in these birds.

An overall high level of sequence similarity was observed between complete *Amazona* mitogenomes. The paucity of polymorphisms in tRNA may indicate conservation of structure and, hence, function. Importantly, the majority of differences within the coding sequence are at the third codon position, leading to synonymous substitutions. The majority of first codon polymorphisms are also synonymous. The presence of an intergenic region between ND5 and CYTB in *A. barbadensis* was due to an insertion of CT creating a premature stop codon. This insertion seems to have occurred after this species diverged from *A. aestiva* and *A. ochrocephala,* since these two species share the same ND5 amino acid sequence, as well as the overlap of the 3’-end of ND5 and the 5’-end of CYTB.

The phylogeny reported here is congruent with a partitioned Bayesian analysis of 117 parrot species based on two mitochondrial genes, two nuclear introns and coded gaps (Schirtzinger *et al.*, 2012). As expected, *A. aestiva* was shown to be a sister group of *A. ochrocephala.*

Analysis of the cytochrome oxidase I (COI) placed the bird, whose mitogenome was described here, close to H_14 from the state of Bahia in Brazil. H_14 contains the majority of COI’s from *A. aestiva* mitogenomes. The H9_clade1_SA, which is the haplotype closest to our sequence by the DNA Surveillance classification (Figure 5), is part of clade 1, which according to Chaves *et al.* (2014), is related to the north-eastern group of Caparroz *et al.* (2009). The latter corresponds to the Brazil states Bahia, Tocantins, Minas Gerais, Distrito Federal, and Goiás. These placements were also confirmed by a phylogenetic tree using the same data. Thus, the specimen whose mitogenome we describe in the present study could be closely related to individuals from the state of Bahia. Also, H9_clade1_SA corresponds to captive *A. aestiva aestiva* specimens, which is consistent with our morphological subspecies classification.

In sum, the mitochondrial genome of the blue-fronted Amazon, *A. aestiva*, was described here for the first time and compared to other whole mitogenomes from the genus *Amazona*. Our data provide a new and high quality mitogenome of an *Amazona* species. Our analyses confirm the presence of conserved sequences and boxes in the duplicated control regions, providing support to previous studies on the organization of this mitogenomic feature. Our data also provide further support for the concerted evolution of these duplicated CRs. The phylogeny confirmed previous findings (e.g., sister relationship between Psittaciformes and Passeriformes, monophyly and basal position of Strigopoidae, monophyly of the Australasian cockatoos, monophyly of subfamily Arinae). Lastly, our data help clarify the haplotype placement of the specimen analyzed, confirming the subspecies classification based on morphological traits.

## Supplementary material

The following online material is available for this article:

Table S1 - Species, NCBI accession IDs, and references of the mitogenomes used.

Table S2 - Mitochondrial genome features of the blue-fronted parrot *Amazona aestiva*.

Table S3 - Sequences analyzed in the taxonomic study.

Table S4 - Mitochondrial gene differences between species.

Table S5 - Polymorphisms found in the whole mitogenome alignment of *Amazona aestiva* and *Amazona barbadensis*.

Table S6 - Polymorphisms found in the whole mitogenome alignment of *Amazona aestiva* and *Amazona ochrocephala*.

Figure S1 - Graph of the sum of adenines and guanines in 45 nucleotide windows

Figure S2 - Photo of individual FVVF132 (“Moisés”) whose mitogenome was analyzed.
