# Supplementary material for: Comparative mitogenomic analyses of Amazona parrots and Psittaciformes
Source: Genet Mol Biol. 2018 Jul-Sep;41(3):593–604. doi: 10.1590/1678-4685-GMB-2017-0023 (PMC6136379; doi:10.1590/1678-4685-GMB-2017-0023)
Supplement: Supplementary file 1 [file 1415-4757-GMB-41-03-2017-0023-20180716-suppl7.pdf]

Supplementary Material to "Comparative mitogenomic analyses of *Amazona* parrots and Psittaciformes"

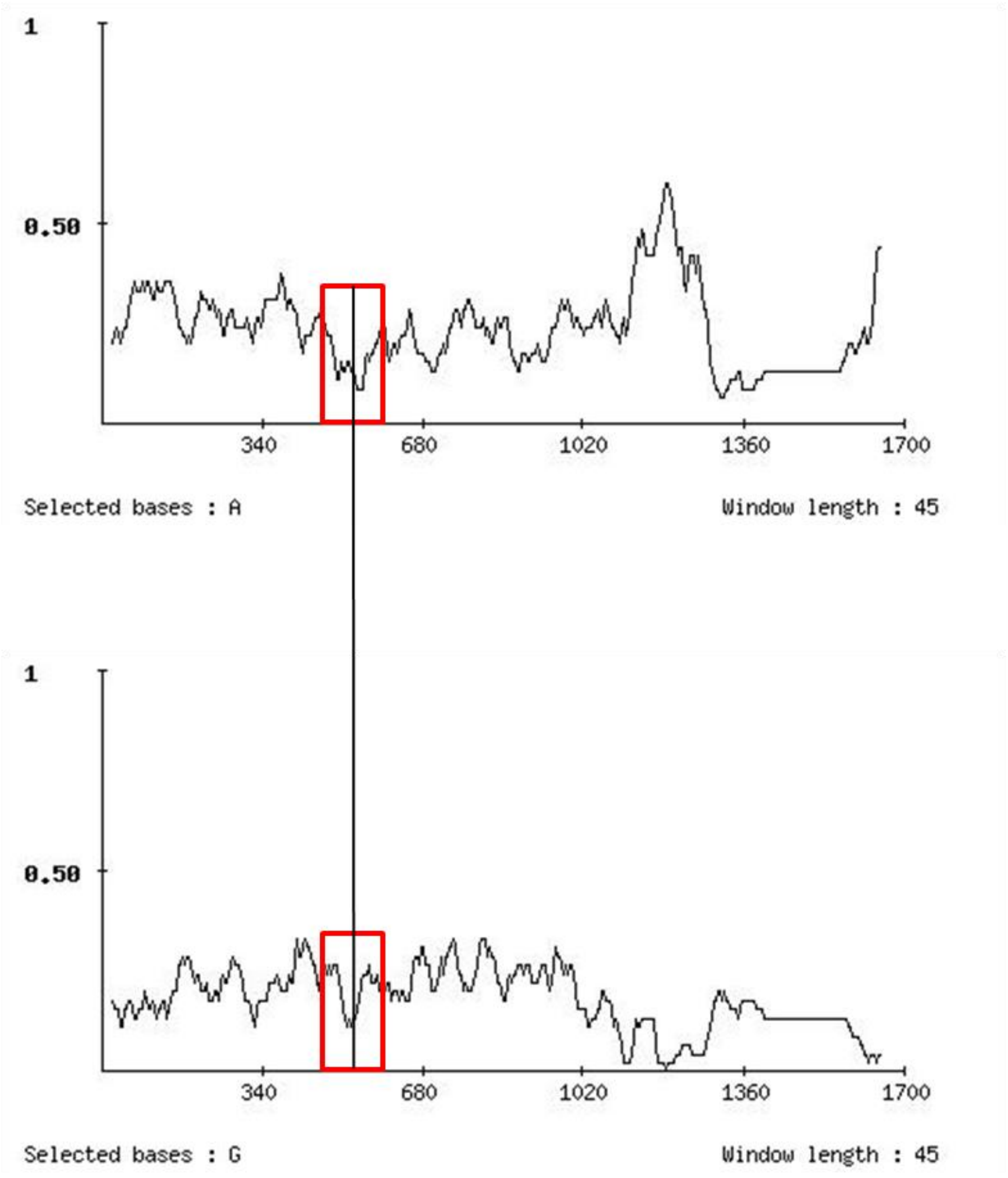

Figure S1 - Graph of the sum of adenines and guanines in 45 nucleotide Windows.
