# Supplementary material for: Comparative mitogenomic analyses of Amazona parrots and Psittaciformes
Source: Genet Mol Biol. 2018 Jul-Sep;41(3):593–604. doi: 10.1590/1678-4685-GMB-2017-0023 (PMC6136379; doi:10.1590/1678-4685-GMB-2017-0023)
Supplement: Supplementary file 2 [file 1415-4757-GMB-41-03-2017-0023-20180716-suppl1.pdf]

# Supplementary Material to "Comparative mitogenomic analyses of *Amazona* parrots and Psittaciformes"

**Table S1** - Species, NCBI accession IDs, and references of the mitogenomes used in the phylogenetic reconstruction.

| NCBI Access ID | Species | Superfamily | Family | Subfamily |
|---|---|---|---|---|
| NC_011708 | *Agapornis roseicollis* | Psittacoidae | Psittaculidae | |
| NC_033336 | *Amazona aestiva* | Psittacoidae | Psittacidae | Arinae |
| JX524615 | *Amazona barbadensis* | Psittacoidae | Psittacidae | Arinae |
| NC_027840 | *Amazona ochrocephala* | Psittacoidae | Psittacidae | Arinae |
| NC_034679 | *Amazona ventralis* | Psittacoidae | Psittacidae | Arinae |
| NC_029319 | *Ara ararauna* | Psittacoidae | Psittacidae | Arinae |
| NC_026029 | *Ara glaucogularis* | Psittacoidae | Psittacidae | Arinae |
| CM002021 | *Ara macao* | Psittacoidae | Psittacidae | Arinae |
| NC_027839 | *Ara militaris* | Psittacoidae | Psittacidae | Arinae |
| JX524613 | *Ara militaris mexicanus* | Psittacoidae | Psittacidae | Arinae |
| KF946546 | *Ara severus* | Psittacoidae | Psittacidae | Arinae |
| JX215256 | *Aratinga mitrata mitrata* | Psittacoidae | Psittacidae | Arinae |
| NC_020325 | *Psittacara acuticaudatus acuticaudatus* | Psittacoidae | Psittacidae | Arinae |
| NC_021764 | *Psittacara [Aratinga] brevipes* | Psittacoidae | Psittacidae | Arinae |
| NC_015197 | *Eupsittula [Aratinga] pertinax* | Psittacoidae | Psittacidae | Arinae |
| NC_026042 | *Psittacara [Aratinga] rubritorquis* | Psittacoidae | Psittacidae | Arinae |
| NC_015530 | *Brotogeris cyanoptera* | Psittacoidae | Psittacidae | Arinae |
| NC_020592 | *Cacatua moluccensis* | Cacatuoidae | | |
| JF414240 | *Cacatua pastinator* | Cacatuoidae | | |
| NC_020594 | *Calyptorhynchus baudinii* | Cacatuoidae | | |
| NC_020593 | *Calyptorhynchus lathami* | Cacatuoidae | | |
| NC_020595 | *Calyptorhynchus latirostris* | Cacatuoidae | | |
| NC_027841 | *Coracopsis vasa* | | Psittrichasiidae | Psittacoidae |
| NC_027842 | *Eclectus roratus* | Psittaculinae | Psittaculidae | Psittacoidae |
| NC_000878 | *Falco peregrinus* | | | |
| NC_027843 | *Forpus passerinus* | Psittacoidae | Psittacidae | Arinae |
| NC_001323 | *Gallus gallus* | | | |
| NC_009134 | *Melopsittacus undulatus* | Psittacoidae | Psittaculidae | |
| NC_027844 | *Myiopsitta monachus* | Psittacoidae | Psittacidae | Arinae |
| NC_019804 | *Neophema chrysogaster* | Psittacoidae | Psittaculidae | |
| NC_027845 | *Nestor notabilis* | Strigopoidae | | |

| NCBI Access ID | Species | Superfamily | Family | Subfamily |
|---|---|---|---|---|
| NC_015192 | *Nymphicus hollandicus* | Cacatuoidae | | |
| KX925978 | *Pionus menstruus menstruus* | | Psittacidae | |
| NC_028404 | *Pyrrhura rupicola* | | Psittacidae | |
| NC_025742 | *Primolius couloni* | Psittacoidae | Psittacidae | Arinae |
| NC_029322 | *Primolius maracana* | Psittacoidae | Psittacidae | Arinae |
| NC_027846 | *Prioniturus luconensis* | Psittaculinae | Psittaculidae | Psittacoidae |
| NC_027847 | *Psittacus erithacus* | Psittacoidae | Psittacidae | Psittacinae |
| NC_027848 | *Psittrichas fulgidus* | | Psittrichasiidae | Psittacoidae |
| NC_031358 | *Psephotellus pulcherrimus* | | Psittaculidae | |
| NC_029161 | *Orthopsittaca manilata* | | Psittacidae | |
| NC_021771 | *Rhynchopsitta terrisi* | Psittacoidae | Psittacidae | Arinae |
| NC_005931 | *Strigops habroptilus* | Strigopoidae | | |
| NC_007897 | *Taeniopygia guttata* | | | |
