# Supplementary material for: Comparative mitogenomic analyses of Amazona parrots and Psittaciformes
Source: Genet Mol Biol. 2018 Jul-Sep;41(3):593–604. doi: 10.1590/1678-4685-GMB-2017-0023 (PMC6136379; doi:10.1590/1678-4685-GMB-2017-0023)
Supplement: Supplementary file 3 [file 1415-4757-GMB-41-03-2017-0023-20180716-suppl3.pdf]

Supplementary Material to “Comparative mitogenomic analyses of *Amazona* parrots and Psittaciformes”

Table S3 - Sequences analyzed in the taxonomic study. Locality code corresponds to localities in Figure 3. Voucher is the identification number in the corresponding collection. Haplotype corresponds to those in Figure 3.

Species	Subspecies	Locality Code	Locality	Voucher	Haplotype	GenBank accession number
A. ochrocephala	*ochrocephala*	**1**	Caicara, Venezuela	AMNH 177109	H_19	AY194400
A. ochrocephala	*ochrocephala*	**2**	Maipures, Venezuela	AMNH 437237	H_20	AY194401
A. ochrocephala	*ochrocephala*	**3**	Carimagua, Colombia	STRI-x-61	H_17	AY194393
A. ochrocephala	*ochrocephala*	**4**	La Macarena, Colombia	ANSP 168178	H_18	AY194402
A. ochrocephala	*ochrocephala*	**5**	Macapá, AP, Brazil	LGEMA 5188	H_5	DQ453631
				LGEMA 5195	H_5	DQ453621
				LGEMA 5197	H_5	DQ453625
				LGEMA 5204	H_5	DQ453607
A. ochrocephala	*xantholaema*	**6**	Ilha do Marajó, PA, Brazil	LGEMA 4663	H_2	DQ453623
				LGEMA 4674	H_3	DQ453626
				LGEMA 5219	H_3	DQ453612
				LGEMA 5221	H_3	DQ453627
				LGEMA 5222	H_3	DQ453604
				STRI-LP1	H_3	AY194378
A. ochrocephala	*ochrocephala*	**7**	Altamira, PA, Brazil	NMNH B06867	H_7	AY194368
				NMNH B07034	H_7	AY194369
A. ochrocephala	*nattereri*	**9**	FLONA Macauã, AC, Brazil	LGEMA 5700	H_6	DQ453616
			Rio Itimarí, AC, Brazil	LGEMA 5716	H_8	DQ453605
A. ochrocephala	*nattereri*	**10**	Assis Brasil, AC, Brazil	LGEMA 5708	H_7	DQ453606
A. ochrocephala	*nattereri*	**11**	Basiléia, AC, Brazil	LGEMA 5706	H_6	DQ453613
			Xapurí, AC, Brazil	LGEMA 5710	H_6	DQ453609
			Pando Department, Bolivia	LGEMA 5707	H_4	DQ453630

Species	Subspecies	Locality Code	Locality	Voucher	Haplotype	GenBank accession number
				LSU B9409	H_6	AY194372
A. ochrocephala	*nattereri*	**12**	Beni, Bolivia	LSU B-25220	H_7	AY194371
A. ochrocephala	*nattereri*	**13**	Santa Cruz Department, Bolivia	LSU B12973	H_16	AY194370
A. aestiva	*xanthopteryx*	**14**	Vila Bela da Santíssima Trindade, MT, Brazil	LGEMA 4951	H_4	DQ453624
				LGEMA 4952	H_4	DQ453615
A. aestiva	[a]	**15**	Provincia del Jujuy, Argentina	UCB 97	H_21	EU340703
				UCB 98	H_7	EU340704
				UCB 99	H_7	EU340705
				UCB 100	H_7	EU340665
				UCB 101	H_21	EU340666
A. aestiva	[a]	**16**	Provincia del Chaco, Argentina	UCB 47	H_7	EU340689
				UCB 48	H_7	EU340690
				UCB 49	H_10	EU340691
				UCB 52	H_7	EU340692
				UCB 55	H_23	EU340693
				UCB 59	H_7	EU340694
				UCB 60	H_7	EU340695
				UCB 61	H_7	EU340696
				UCB 64	H_10	EU340697
				UCB 67	H_7	EU340698
				UCB 70	H_7	EU340699
				UCB 74	H_24	EU340700
				UCB 77	H_7	EU340701
				UCB 86	H_7	EU340702
A. aestiva	*aestiva*	**17**	Miranda, MS, Brazil	LGEMA 5812	H_3	DQ453611
				LGEMA 5813	H_7	EU340629
				LGEMA 5814	H_9	EU340630
				LGEMA 5815	H_9	DQ453608
				LGEMA 5816	H_10	EU340631
				LGEMA 5818	H_7	DQ453610
				LGEMA 5820	H_7	EU340633
				LGEMA 5821	H_7	DQ453617
				LGEMA 5822	H_7	EU340634
				LGEMA 5824	H_9	DQ453628
				LGEMA 5825	H_11	EU340636
				LGEMA 5827	H_7	EU340637
				LGEMA 5828	H_7	EU340638
				LGEMA 5831	H_3	EU340639

Species	Subspecies	Locality Code	Locality	Voucher	Haplotype	GenBank accession number
				LGEMA 5833	H_7	EU340640
A. aestiva	[a]	**18**	Parque Nacional das Emas, GO, Brazil	UCB 104	H_3	EU340667
				UCB 107	H_3	EU340668
				UCB 152	H_3	EU340674
A. aestiva	[a]	**19**	Distrito Federal, DF, Brazil	UCB 02	H_3	EU340658
				UCB 03	H_3	EU340659
				UCB 04	H_3	EU340660
				UCB 05	H_3	EU340661
				UCB 06	H_3	EU340662
				UCB 07	H_3	EU340663
				UCB 09	H_3	EU340664
				UCB 11	H_3	EU340670
				UCB 109	H_3	EU340669
				UCB 115	H_3	EU340671
				UCB 141	H_3	EU340672
				UCB 143	H_3	EU340673
A. aestiva	*aestiva*	**20**	Parque Nacional Grande Sertão Veredas, MG, Brazil	LGEMA 7683	H_3	EU340641
A. ochrocephala	*ochrocephala*[b]			LGEMA 7684	H_7	DQ453619
				LGEMA 7685	H_3	DQ453621[c]
				LGEMA 7686	H_3	EU340644
				LGEMA 7687	H_12	DQ453620
				LGEMA 7688	H_3	DQ453622
				LGEMA 7689	H_3	DQ453618
				LGEMA 7690	H_3	EU340648
				LGEMA 7691	H_13	EU340649
				LGEMA 7692	H_3	EU340650
A. aestiva	[a]	**21**	Gurupi, TO, Brazil	UCB 155	H_3	EU340675
				UCB 157	H_3	EU340676
				UCB 158	H_3	EU340677
				UCB 159	H_3	EU340678
				UCB 160	H_3	EU340679
				UCB 161	H_22	EU340680
				UCB 162	H_3	EU340681
				UCB 163	H_3	EU340682
				UCB 164	H_3	EU340683
				UCB 165	H_3	EU340684
				UCB 166	H_3	EU340685
				UCB 167	H_3	EU340686

Species	Subspecies	Locality Code	Locality	Voucher	Haplotype	GenBank accession number
				UCB 168	H_3	EU340687
				UCB 169	H_3	EU340688
A. aestiva	*	**22**	Feira de Santana, BA, Brazil	LGEMA 4374	H_14	EU340651
				LGEMA 4375	H_3	EU340652
				LGEMA 4376	H_3	EU340653
				LGEMA 4377	H_3	EU340654
				LGEMA 4378	H_15	EU340655
				LGEMA 4379	H_3	EU340656
				LGEMA 4380	H_14	EU340657

[a] Data from Caparroz,R., Seixas,G.H.F., Berkunsky,I. and Collevatti,R.G. Recent evolutionary history of the blue-fronted amazon (Amazona aestiva) and A. ochrocephala complex (Psittaciformes: Aves): implications of species limits and conservation. *Unpublished*. No subspecies specification on GenBank record.

[b] One specimen (voucher LGEMA7685, GenBank DQ453621).

[C] This ID refers to a *Amazona ochrocephala ochrocephala*.
