# Supplementary material for: Comparative mitogenomic analyses of Amazona parrots and Psittaciformes
Source: Genet Mol Biol. 2018 Jul-Sep;41(3):593–604. doi: 10.1590/1678-4685-GMB-2017-0023 (PMC6136379; doi:10.1590/1678-4685-GMB-2017-0023)
Supplement: Supplementary file 4 [file 1415-4757-GMB-41-03-2017-0023-20180716-suppl2.pdf]

Supplementary Material to "Comparative mitogenomic analyses of *Amazona* parrots and Psittaciformes"

Table S2 -Mitochondrial genome features of the Blue-fronted Parrot *Amazona aestiva*.

Gene	Position		Size		Codons		Start Amino Acid	Anti-Codon	Strand
	Start	End	Nucleotide	Amino Acids	Start	Stop			
tRNA-Phe	1	66	66	-	-	-	-	GAA	L
12S rRNA	66	1033	968	-	-	-	-	-	L
tRNA-Val	1033	1104	72	-	-	-	-	TAC	L
16S rRNA	1106	2673	1568	-	-	-	-	-	L
tRNA-Leu	2673	2747	75	-	-	-	-	TAA	L
ND1	2754	3734	980	326	atg	ag*/ag(a)	M	-	L
tRNA-Ile	3733	3804	72	-	-	-	-	GAT	L
tRNA-Gln	3882	3812	71	-	-	-	-	TTG	H
tRNA-Met	3882	3950	69	-	-	-	-	CAT	L
ND2	3951	4991	1041	346	ata	taa	M	-	L
tRNA-Trp	4991	5060	70	-	-	-	-	TCA	L
tRNA-Ala	5130	5062	69	-	-	-	-	TGC	H
tRNA-Asn	5205	5132	74	-	-	-	-	GTT	H
tRNA-Cys	5274	5208	67	-	-	-	-	GCA	H
tRNA-Tyr	5344	5274	71	-	-	-	-	GTA	H
COX1	5354	6901	1548	515	gtg	agg	V	-	L
tRNA-Ser	6968	6893	76	-	-	-	-	TGA	H

Gene	Position		Size		Codons		Start Amino Acid	Anti-Codon	Strand
	Start	End	Nucleotide	Amino Acids	Start	Stop			
tRNA-Asp	6971	7039	69	-	-	-	-	GTC	L
COX2	7042	7725	684	227	atg	taa	M	-	L
tRNA- Lys	7727	7795	69	-	-	-	-	TTT	L
ATP8	7797	7964	168	55	atg	taa	M	-	L
ATP6	7955	8638	684	227	atg	taa	M	-	L
COX3	8638	9421	784	261	atg	t**/t(a)(a)	M	-	L
tRNA-Gly	9422	9489	68	-	-	-	-	TCC	L
ND3	9490	9841	352	117	ata	taa	M	-	L
tRNA-Arg	9841	9909	69	-	-	-	-	TCG	L
ND4L	9911	10207	297	98	atg	taa	M	-	L
ND4	10201	11593	1393	464	atg	t**/t(a)(a)	M	-	L
tRNA-His	11594	11662	69	-	-	-	-	GTG	L
tRNA-Ser	11663	11728	66	-	-	-	-	GCU	L
tRNA-Leu	11728	11798	71	-	-	-	-	TAG	L
ND5	11799	13622	1824	607	gtg	taa	V	-	L
CYTB	13622	14761	1140	379	atg	taa	M	-	L
tRNA-Thr	14762	14830	69	-	-	-	-	TGT	L
pseudo ND6	14831	14890	60	-		-	-	-	L
pseudotRNA-Glu	14891	14956	66	-	-	-	-	-	L
Control Region 1	14957	16521	1565	-	-	-	-	-	L
tRNA-Pro	16590	16522	69	-	-	-	-	TGG	H
ND6	17112	16594	519	172	atg	ag*/ag(a)	M	-	H
tRNA-Glu	17182	17114	69	-	-	-	-	TTC	H
Control Region 2	17183	18853	1671	-		-	-	-	L
