# Supplementary material for: Comparative mitogenomic analyses of Amazona parrots and Psittaciformes
Source: Genet Mol Biol. 2018 Jul-Sep;41(3):593–604. doi: 10.1590/1678-4685-GMB-2017-0023 (PMC6136379; doi:10.1590/1678-4685-GMB-2017-0023)
Supplement: Table S4 - [file 1415-4757-GMB-41-03-2017-0023-20180716-suppl4.pdf]

Supplementary Material to “Comparative mitogenomic analyses of *Amazona* parrots and Psittaciformes”

Table S4 - Mitochondrial gene differences between species. AaesXAbar shows the comparison between *Amazona aestiva* and *Amazona barbadensis*. AaesXAbar shows the comparison of *Amazona aestiva* and *Amazona ochrocephala*.

ATP6

**AaesXAbar**							**AaesXAoch**						
	**Codon Position 3**	**14**	**INDEL**	**0**	**Synonymous**	**17**		**Codon Position 3**	**4**	**INDEL**	**0**	**Synonymous**	**6**
	**Codon Position 2**	**0**	**Transitions**	**17**	**Missense**	**1**		**Codon Position 2**	**0**	**Transitions**	**6**	**Missense**	**0**
	**Codon Position 1**	**4**	**Transversions**	**1**	**Nonsense**	**0**		**Codon Position 1**	**2**	**Transversions**	**0**	**Nonsense**	**0**

ATP8

**AaesXAbar**	**AaesXAoch**

	Codon Position 3	0	INDEL	0	Synonymous	0		Codon Position 3	1	INDEL	0	Synonymous	1
	Codon Position 2	1	Transitions	1	Missense	1		Codon Position 2	1	Transitions	2	Missense	1
	Codon Position 1	0	Transversions	0	Nonsense	0		Codon Position 1	0	Transversions	0	Nonsense	0
**COX1**													
**AaesXAbar**							**AaesXAoch**						
	Codon Position 3	23	INDEL	0	Synonymous	25		Codon Position 3	8	INDEL	0	Synonymous	8
	Codon Position 2	1	Transitions	26	Missense	1		Codon Position 2	0	Transitions	8	Missense	0
	Codon Position 1	2	Transversions	0	Nonsense	0		Codon Position 1	0	Transversions	0	Nonsense	0
**COX2**													
**AaesXAbar**							**AaesXAoch**						
	Codon Position 3	12	INDEL	0	Synonymous	12		Codon Position 3	3	INDEL	0	Synonymous	3
	Codon Position 2	2	Transitions	15	Missense	3		Codon Position 2	0	Transitions	4	Missense	1
	Codon Position 1	1	Transversions	0	Nonsense	0		Codon Position 1	1	Transversions	0	Nonsense	0
**COX3**													
**AaesXAbar**							**AaesXAoch**						
	Codon Position 3	15	INDEL	0	Synonymous	16		Codon Position 3	7	INDEL	0	Synonymous	7

	**Codon Position 2**	**1**	**Transitions**	**17**	**Missense**	**2**		**Codon Position 2**	**1**	**Transitions**	**8**	**Missense**	**1**
	**Codon Position 1**	**2**	**Transversions**	**1**	**Nonsense**	**0**		**Codon Position 1**	**0**	**Transversions**	**0**	**Nonsense**	**0**
**CYTB**													
**AaesXAbar**							**AaesXAoch**						
	**Codon Position 3**	**34**	**INDEL**	**0**	**Synonymous**	**36**		**Codon Position 3**	**9**	**INDEL**	**0**	**Synonymous**	**9**
	**Codon Position 2**	**0**	**Transitions**	**40**	**Missense**	**4**		**Codon Position 2**	**1**	**Transitions**	**9**	**Missense**	**0**
	**Codon Position 1**	**6**	**Transversions**	**0**	**Nonsense**	**0**		**Codon Position 1**	**0**	**Transversions**	**0**	**Nonsense**	**0**
**ND1**													
**AaesXAbar**							**AaesXAoch**						
	**Codon Position 3**	**15**	**INDEL**	**0**	**Synonymous**	**15**		**Codon Position 3**	**4**	**INDEL**	**0**	**Synonymous**	**4**
	**Codon Position 2**	**0**	**Transitions**	**15**	**Missense**	**1**		**Codon Position 2**	**0**	**Transitions**	**4**	**Missense**	**0**
	**Codon Position 1**	**1**	**Transversions**	**1**	**Nonsense**	**0**		**Codon Position 1**	**0**	**Transversions**	**0**	**Nonsense**	**0**
**ND2**													
**AaesXAbar**							**AaesXAoch**						
	**Codon Position 3**	**10**	**INDEL**	**0**	**Synonymous**	**11**		**Codon Position 3**	**4**	**INDEL**	**0**	**Synonymous**	**4**

	**Codon Position 2**	**1**	**Transitions**	**14**	**Missense**	**4**		**Codon Position 2**	**2**	**Transitions**	**7**	**Missense**	**3**
	**Codon Position 1**	**4**	**Transversions**	**1**	**Nonsense**	**0**		**Codon Position 1**	**1**	**Transversions**	**0**	**Nonsense**	**0**
**ND3**													
**AaesXAbar**							**AaesXAoch**						
	**Codon Position 3**	**4**	**INDEL**	**0**	**Synonymous**	**4**		**Codon Position 3**	**2**	**INDEL**	**0**	**Synonymous**	**2**
	**Codon Position 2**	**0**	**Transitions**	**4**	**Missense**	**0**		**Codon Position 2**	**0**	**Transitions**	**2**	**Missense**	**0**
	**Codon Position 1**	**0**	**Transversions**	**0**	**Nonsense**	**0**		**Codon Position 1**	**0**	**Transversions**	**0**	**Nonsense**	**0**
**ND4**													
**AaesXAbar**							**AaesXAoch**						
	**Codon Position 3**	**20**	**INDEL**	**0**	**Synonymous**	**25**		**Codon Position 3**	**5**	**INDEL**	**0**	**Synonymous**	**6**
	**Codon Position 2**	**1**	**Transitions**	**28**	**Missense**	**4**		**Codon Position 2**	**1**	**Transitions**	**9**	**Missense**	**3**
	**Codon Position 1**	**8**	**Transversions**	**1**	**Nonsense**	**0**		**Codon Position 1**	**3**	**Transversions**	**0**	**Nonsense**	**0**
**ND4L**													
**AaesXAbar**							**AaesXAoch**						
	**Codon Position 3**	**5**	**INDEL**	**0**	**Synonymous**	**6**		**Codon Position 3**	**1**	**INDEL**	**0**	**Synonymous**	**1**
	**Codon Position 2**	**0**	**Transitions**	**7**	**Missense**	**2**		**Codon Position 2**	**0**	**Transitions**	**2**	**Missense**	**1**
	**Codon Position 1**	**3**	**Transversions**	**1**	**Nonsense**	**0**		**Codon Position 1**	**1**	**Transversions**	**0**	**Nonsense**	**0**

**ND5**													
**AaesXAbar**							**AaesXAoch**						
	**Codon Position 3**	**42**	**INDEL**	**3**	**Synonymous**	**45**		**Codon Position 3**	**14**	**INDEL**	**0**	**Synonymous**	**15**
	**Codon Position 2**	**5**	**Transitions**	**55**	**Missense**	**14**		**Codon Position 2**	**1**	**Transitions**	**17**	**Missense**	**2**
	**Codon Position 1**	**12**	**Transversions**	**1**	**Nonsense**	**0**		**Codon Position 1**	**2**	**Transversions**	**0**	**Nonsense**	**0**
**ND6**													
**AaesXAbar**							**AaesXAoch**						
	**Codon Position 3**	**13**	**INDEL**	**0**	**Synonymous**	**14**		**Codon Position 3**	**8**	**INDEL**	**0**	**Synonymous**	**8**
	**Codon Position 2**	**4**	**Transitions**	**22**	**Missense**	**9**		**Codon Position 2**	**1**	**Transitions**	**9**	**Missense**	**4**
	**Codon Position 1**	**6**	**Transversions**	**1**	**Nonsense**	**0**		**Codon Position 1**	**4**	**Transversions**	**3**	**Nonsense**	**0**
**s-rRNA**													
**AaesXAbar**							**AaesXAoch**						
	**Codon Position 3**	**0**	**INDEL**	**0**	**Synonymous**	**0**		**Codon Position 3**	**0**	**INDEL**	**0**	**Synonymous**	**0**
	**Codon Position 2**	**0**	**Transitions**	**8**	**Missense**	**0**		**Codon Position 2**	**0**	**Transitions**	**1**	**Missense**	**0**
	**Codon Position 1**	**0**	**Transversions**	**2**	**Nonsense**	**0**		**Codon Position 1**	**0**	**Transversions**	**1**	**Nonsense**	**0**
**l-rRNA**													

**AaesXAbar**							**AaesXAoch**						
	**Codon Position 3**	**0**	**INDEL**	**2**	**Synonymous**	**0**		**Codon Position 3**	**0**	**INDEL**	**1**	**Synonymous**	**0**
	**Codon Position 2**	**0**	**Transitions**	**20**	**Missense**	**0**		**Codon Position 2**	**0**	**Transitions**	**6**	**Missense**	**0**
	**Codon Position 1**	**0**	**Transversions**	**0**	**Nonsense**	**0**		**Codon Position 1**	**0**	**Transversions**	**0**	**Nonsense**	**0**
**tRNA-Arg**													
**AaesXAbar**							**AaesXAoch**						
	**Codon Position 3**	**0**	**INDEL**	**0**	**Synonymous**	**0**		**Codon Position 3**	**0**	**INDEL**	**0**	**Synonymous**	**0**
	**Codon Position 2**	**0**	**Transitions**	**1**	**Missense**	**0**		**Codon Position 2**	**0**	**Transitions**	**0**	**Missense**	**0**
	**Codon Position 1**	**0**	**Transversions**	**0**	**Nonsense**	**0**		**Codon Position 1**	**0**	**Transversions**	**0**	**Nonsense**	**0**
**tRNA-Asp**													
**AaesXAbar**							**AaesXAoch**						
	**Codon Position 3**	**0**	**INDEL**	**0**	**Synonymous**	**0**		**Codon Position 3**	**0**	**INDEL**	**0**	**Synonymous**	**0**
	**Codon Position 2**	**0**	**Transitions**	**2**	**Missense**	**0**		**Codon Position 2**	**0**	**Transitions**	**1**	**Missense**	**0**
	**Codon Position 1**	**0**	**Transversions**	**0**	**Nonsense**	**0**		**Codon Position 1**	**0**	**Transversions**	**0**	**Nonsense**	**0**
**tRNA-His**													
**AaesXAbar**							**AaesXAoch**						
	**Codon Position 3**	**0**	**INDEL**	**0**	**Synonymous**	**0**		**Codon Position 3**	**0**	**INDEL**	**0**	**Synonymous**	**0**
	**Codon Position 2**	**0**	**Transitions**	**1**	**Missense**	**0**		**Codon Position 2**	**0**	**Transitions**	**0**	**Missense**	**0**

	Codon Position 1	0	Transversions	0	Nonsense	0		Codon Position 1	0	Transversions	0	Nonsense	0
**tRNA-Ile**													
AaesXAbar							AaesXAoch						
	Codon Position 3	0	INDEL	0	Synonymous	0		Codon Position 3	0	INDEL	0	Synonymous	0
	Codon Position 2	0	Transitions	1	Missense	0		Codon Position 2	0	Transitions	0	Missense	0
	Codon Position 1	0	Transversions	0	Nonsense	0		Codon Position 1	0	Transversions	0	Nonsense	0
**tRNA-Lys**													
AaesXAbar							AaesXAoch						
	Codon Position 3	0	INDEL	0	Synonymous	0		Codon Position 3	0	INDEL	0	Synonymous	0
	Codon Position 2	0	Transitions	1	Missense	0		Codon Position 2	0	Transitions	0	Missense	0
	Codon Position 1	0	Transversions	0	Nonsense	0		Codon Position 1	0	Transversions	0	Nonsense	0
**tRNA-Phe**													
AaesXAbar							AaesXAoch						
	Codon Position 3	0	INDEL	1	Synonymous	0		Codon Position 3	0	INDEL	0	Synonymous	0
	Codon Position 2	0	Transitions	2	Missense	0		Codon Position 2	0	Transitions	0	Missense	0
	Codon Position 1	0	Transversions	0	Nonsense	0		Codon Position 1	0	Transversions	0	Nonsense	0
**tRNA-Pro**													
AaesXAbar							AaesXAoch						
	Codon Position 3	0	INDEL	0	Synonymous	0		Codon Position 3	0	INDEL	1	Synonymous	0

	Codon Position 2	0	Transitions	1	Missense	0		Codon Position 2	0	Transitions	1	Missense	0
	Codon Position 1	0	Transversions	0	Nonsense	0		Codon Position 1	0	Transversions	0	Nonsense	0
Between tRNA-Pro and ND6													
AaesXAbar							AaesXAoch						
	Codon Position 3	0	INDEL	0	Synonymous	0		Codon Position 3	1	INDEL	0	Synonymous	1
	Codon Position 2	1	Transitions	1	Missense	1		Codon Position 2	1	Transitions	2	Missense	1
	Codon Position 1	0	Transversions	0	Nonsense	0		Codon Position 1	0	Transversions	0	Nonsense	0
pseudoND6													
AaesXAbar							AaesXAoch						
	Codon Position 3	0	INDEL	1	Synonymous	0		Codon Position 3	0	INDEL	0	Synonymous	0
	Codon Position 2	0	Transitions	1	Missense	0		Codon Position 2	0	Transitions	2	Missense	0
	Codon Position 1	0	Transversions	0	Nonsense	0		Codon Position 1	0	Transversions	0	Nonsense	0
pseudo-tRNA													
AaesXAbar							AaesXAoch						
	Codon Position 3	0	INDEL	1	Synonymous	0		Codon Position 3	0	INDEL	0	Synonymous	0
	Codon Position 2	0	Transitions	2	Missense	0		Codon Position 2	0	Transitions	0	Missense	0
	Codon Position 1	0	Transversions	0	Nonsense	0		Codon Position 1	0	Transversions	0	Nonsense	0

**Control Region1**												
**AaesXAbar**							**AaesXAoch**					
	**Codon Position 3**	**0**	**INDEL**	**69**	**Synonymous**	**0**		**Codon Position 3**	**0**	**INDEL**	**30**	**Synonymous**	**0**
	**Codon Position 2**	**0**	**Transitions**	**60**	**Missense**	**0**		**Codon Position 2**	**0**	**Transitions**	**30**	**Missense**	**0**
	**Codon Position 1**	**0**	**Transversions**	**10**	**Nonsense**	**0**		**Codon Position 1**	**0**	**Transversions**	**2**	**Nonsense**	**0**
**Control Region2**												
**AaesXAbar**							**AaesXAoch**					
	**Codon Position 3**	**0**	**INDEL**	**14**	**Synonymous**	**0**		**Codon Position 3**	**0**	**INDEL**	**35**	**Synonymous**	**0**
	**Codon Position 2**	**0**	**Transitions**	**43**	**Missense**	**0**		**Codon Position 2**	**0**	**Transitions**	**30**	**Missense**	**0**
	**Codon Position 1**	**0**	**Transversions**	**7**	**Nonsense**	**0**		**Codon Position 1**	**0**	**Transversions**	**3**	**Nonsense**	**0**
