# Supplementary material for: Comparative mitogenomic analyses of Amazona parrots and Psittaciformes
Source: Genet Mol Biol. 2018 Jul-Sep;41(3):593–604. doi: 10.1590/1678-4685-GMB-2017-0023 (PMC6136379; doi:10.1590/1678-4685-GMB-2017-0023)
Supplement: Table S5 - [file 1415-4757-GMB-41-03-2017-0023-20180716-suppl5.pdf]

Supplementary Material to "Comparative mitogenomic analyses of *Amazona* parrots and Psittaciformes"

Table S5 - Polymorphisms found in the whole mitogenome alignment of *Amazona aestiva* and *Amazona barbadensis*.

Genbank ID	Organism	Gene	Type	Start	Actual Position	End	Strand	Codon Position	A. aestiva	A. barbadensis	Type of Mutation	A. aestiva AA	A. barbadensis AA	Type of Mutation	Product
KT361659	Amazona aestiva	tRNA-Phe	tRNA	1	39	66	+		C	T	transition				transfer RNA Phenilalanine
KT361659	Amazona aestiva	tRNA-Phe	tRNA	1	45	66	+		T	C	transition				transfer RNA Phenilalanine
KT361659	Amazona aestiva	tRNA-Phe	tRNA	1	50	66	+		.	T	INDEL				transfer RNA Phenilalanine
KT361659	Amazona aestiva	s-rRNA	rRNA	66	146	1033	+		A	T	transversion				12S ribosomal RNA
KT361659	Amazona aestiva	s-rRNA	rRNA	66	174	1033	+		G	A	transition				12S ribosomal RNA
KT361659	Amazona aestiva	s-rRNA	rRNA	66	206	1033	+		C	T	transition				12S ribosomal RNA
KT361659	Amazona aestiva	s-rRNA	rRNA	66	277	1033	+		C	T	transition				12S ribosomal RNA
KT361659	Amazona aestiva	s-rRNA	rRNA	66	281	1033	+		G	A	transition				12S ribosomal RNA
KT361659	Amazona aestiva	s-rRNA	rRNA	66	284	1033	+		A	G	transition				12S ribosomal RNA
KT361659	Amazona aestiva	s-rRNA	rRNA	66	348	1033	+		G	A	transition				12S ribosomal RNA
KT361659	Amazona aestiva	s-rRNA	rRNA	66	428	1033	+		A	C	transversion				12S ribosomal RNA
KT361659	Amazona aestiva	s-rRNA	rRNA	66	944	1033	+		T	C	transition				12S ribosomal RNA
KT361659	Amazona aestiva	s-rRNA	rRNA	66	955	1033	+		A	G	transition				12S ribosomal RNA
KT361659	Amazona aestiva	l-rRNA	rRNA	1106	1131	2673	+		C	T	transition				16S ribosomal RNA
KT361659	Amazona aestiva	l-rRNA	rRNA	1106	1332	2673	+		A	G	transition				16S ribosomal RNA
KT361659	Amazona aestiva	l-rRNA	rRNA	1106	1485	2673	+		A	G	transition				16S ribosomal RNA
KT361659	Amazona aestiva	l-rRNA	rRNA	1106	1613	2673	+		T	C	transition				16S ribosomal RNA
KT361659	Amazona aestiva	l-rRNA	rRNA	1106	1648	2673	+		T	C	transition				16S ribosomal RNA
KT361659	Amazona aestiva	l-rRNA	rRNA	1106	1674	2673	+		A	G	transition				16S ribosomal RNA
KT361659	Amazona aestiva	l-rRNA	rRNA	1106	1676	2673	+		.	A	INDEL				16S ribosomal RNA
KT361659	Amazona aestiva	l-rRNA	rRNA	1106	1676	2673	+		.	A	INDEL				16S ribosomal RNA
KT361659	Amazona aestiva	l-rRNA	rRNA	1106	1684	2673	+		T	C	transition				16S ribosomal RNA
KT361659	Amazona aestiva	l-rRNA	rRNA	1106	1777	2673	+		C	T	transition				16S ribosomal RNA
KT361659	Amazona aestiva	l-rRNA	rRNA	1106	1797	2673	+		C	T	transition				16S ribosomal RNA
KT361659	Amazona aestiva	l-rRNA	rRNA	1106	2082	2673	+		A	G	transition				16S ribosomal RNA
KT361659	Amazona aestiva	l-rRNA	rRNA	1106	2138	2673	+		A	G	transition				16S ribosomal RNA
KT361659	Amazona aestiva	l-rRNA	rRNA	1106	2189	2673	+		T	C	transition				16S ribosomal RNA
KT361659	Amazona aestiva	l-rRNA	rRNA	1106	2236	2673	+		T	C	transition				16S ribosomal RNA
KT361659	Amazona aestiva	l-rRNA	rRNA	1106	2318	2673	+		T	C	transition				16S ribosomal RNA
KT361659	Amazona aestiva	l-rRNA	rRNA	1106	2323	2673	+		T	C	transition				16S ribosomal RNA
KT361659	Amazona aestiva	l-rRNA	rRNA	1106	2392	2673	+		T	C	transition				16S ribosomal RNA

Genbank ID	Organism	Gene	Type	Start	Actual Position	End	Strand	Codon Position	A. aestiva	A. barbadensis	Type of Mutation	A. aestiva AA	A. barbadensis AA	Type of Mutation	Product
KT361659	Amazona aestiva	l-rRNA	rRNA	1106	2614	2673	+		C	T	transition				16S ribosomal RNA
KT361659	Amazona aestiva	l-rRNA	rRNA	1106	2637	2673	+		G	A	transition				16S ribosomal RNA
KT361659	Amazona aestiva	l-rRNA	rRNA	1106	2647	2673	+		T	C	transition				16S ribosomal RNA
KT361659	Amazona aestiva	l-rRNA	rRNA	1106	2654	2673	+		T	C	transition				16S ribosomal RNA
KT361659	Amazona aestiva	ND1	CDS	2754	2810	3733	+	3	C	T	transition	I	I	synonymous	NADH dehydrogenase subunit 1
KT361659	Amazona aestiva	ND1	CDS	2754	2816	3733	+	3	C	T	transition	I	I	synonymous	NADH dehydrogenase subunit 1
KT361659	Amazona aestiva	ND1	CDS	2754	2912	3733	+	3	A	G	transition	L	L	synonymous	NADH dehydrogenase subunit 1
KT361659	Amazona aestiva	ND1	CDS	2754	2960	3733	+	3	T	C	transition	I	I	synonymous	NADH dehydrogenase subunit 1
KT361659	Amazona aestiva	ND1	CDS	2754	2981	3733	+	3	G	A	transition	P	P	synonymous	NADH dehydrogenase subunit 1
KT361659	Amazona aestiva	ND1	CDS	2754	2987	3733	+	3	G	C	transversion	L	L	synonymous	NADH dehydrogenase subunit 1
KT361659	Amazona aestiva	ND1	CDS	2754	3030	3733	+	1	G	A	transition	V	I	missense	NADH dehydrogenase subunit 1
KT361659	Amazona aestiva	ND1	CDS	2754	3110	3733	+	3	G	A	transition	L	L	synonymous	NADH dehydrogenase subunit 1
KT361659	Amazona aestiva	ND1	CDS	2754	3113	3733	+	3	A	G	transition	A	A	synonymous	NADH dehydrogenase subunit 1
KT361659	Amazona aestiva	ND1	CDS	2754	3185	3733	+	3	G	A	transition	V	V	synonymous	NADH dehydrogenase subunit 1
KT361659	Amazona aestiva	ND1	CDS	2754	3287	3733	+	3	G	A	transition	E	E	synonymous	NADH dehydrogenase subunit 1
KT361659	Amazona aestiva	ND1	CDS	2754	3377	3733	+	3	T	C	transition	L	L	synonymous	NADH dehydrogenase subunit 1
KT361659	Amazona aestiva	ND1	CDS	2754	3383	3733	+	3	G	A	transition	E	E	synonymous	NADH dehydrogenase subunit 1
KT361659	Amazona aestiva	ND1	CDS	2754	3473	3733	+	3	A	G	transition	M	M	synonymous	NADH dehydrogenase subunit 1
KT361659	Amazona aestiva	ND1	CDS	2754	3554	3733	+	3	A	G	transition	L	L	synonymous	NADH dehydrogenase subunit 1
KT361659	Amazona aestiva	ND1	CDS	2754	3614	3733	+	3	G	A	transition	R	R	synonymous	NADH dehydrogenase subunit 1
KT361659	Amazona aestiva	tRNA-Ile	tRNA	3733	3751	3804	+		T	C	transition				transfer RNA isoleucine
KT361659	Amazona aestiva	ND2	CDS	3951	3976	4991	+	2	C	T	transition	S	L	missense	NADH dehydrogenase subunit 2
KT361659	Amazona aestiva	ND2	CDS	3951	4019	4991	+	3	C	T	transition	S	S	synonymous	NADH dehydrogenase subunit 2
KT361659	Amazona aestiva	ND2	CDS	3951	4076	4991	+	3	T	C	transition	P	P	synonymous	NADH dehydrogenase subunit 2
KT361659	Amazona aestiva	ND2	CDS	3951	4193	4991	+	3	A	G	transition	T	T	synonymous	NADH dehydrogenase subunit 2
KT361659	Amazona aestiva	ND2	CDS	3951	4199	4991	+	3	A	G	transition	Q	Q	synonymous	NADH dehydrogenase subunit 2
KT361659	Amazona aestiva	ND2	CDS	3951	4202	4991	+	3	A	G	transition	W	W	synonymous	NADH dehydrogenase subunit 2
KT361659	Amazona aestiva	ND2	CDS	3951	4209	4991	+	1	A	G	transition	T	A	missense	NADH dehydrogenase subunit 2
KT361659	Amazona aestiva	ND2	CDS	3951	4226	4991	+	3	C	T	transition	P	P	synonymous	NADH dehydrogenase subunit 2
KT361659	Amazona aestiva	ND2	CDS	3951	4434	4991	+	1	A	G	transition	I	V	missense	NADH dehydrogenase subunit 2
KT361659	Amazona aestiva	ND2	CDS	3951	4544	4991	+	3	C	T	transition	P	P	synonymous	NADH dehydrogenase subunit 2
KT361659	Amazona aestiva	ND2	CDS	3951	4548	4991	+	1	C	T	transition	L	L	synonymous	NADH dehydrogenase subunit 2
KT361659	Amazona aestiva	ND2	CDS	3951	4694	4991	+	3	A	T	transversion	L	L	synonymous	NADH dehydrogenase subunit 2
KT361659	Amazona aestiva	ND2	CDS	3951	4757	4991	+	3	G	A	transition	E	E	synonymous	NADH dehydrogenase subunit 2
KT361659	Amazona aestiva	ND2	CDS	3951	4803	4991	+	1	G	A	transition	A	T	missense	NADH dehydrogenase subunit 2
KT361659	Amazona aestiva	ND2	CDS	3951	4967	4991	+	3	T	C	transition	S	S	synonymous	NADH dehydrogenase subunit 2
KT361659	Amazona aestiva	COX1	CDS	5354	5361	6901	+	2	C	T	transition	T	M	missense	cytochrome c oxidase subunit I
KT361659	Amazona aestiva	COX1	CDS	5354	5443	6901	+	3	C	T	transition	G	G	synonymous	cytochrome c oxidase subunit I
KT361659	Amazona aestiva	COX1	CDS	5354	5455	6901	+	3	T	C	transition	S	S	synonymous	cytochrome c oxidase subunit I
KT361659	Amazona aestiva	COX1	CDS	5354	5456	6901	+	1	C	T	transition	L	L	synonymous	cytochrome c oxidase subunit I
KT361659	Amazona aestiva	COX1	CDS	5354	5596	6901	+	3	G	A	transition	W	W	synonymous	cytochrome c oxidase subunit I

Genbank ID	Organism	Gene	Type	Start	Actual Position	End	Strand	Codon Position	A. aestiva	A. barbadensis	Type of Mutation	A. aestiva AA	A. barbadensis AA	Type of Mutation	Product
KT361659	Amazona aestiva	COX1	CDS	5354	5710	6901	+	3	G	A	transition	E	E	synonymous	cytochrome c oxidase subunit I
KT361659	Amazona aestiva	COX1	CDS	5354	5809	6901	+	3	G	A	transition	L	L	synonymous	cytochrome c oxidase subunit I
KT361659	Amazona aestiva	COX1	CDS	5354	5950	6901	+	3	G	A	transition	L	L	synonymous	cytochrome c oxidase subunit I
KT361659	Amazona aestiva	COX1	CDS	5354	5965	6901	+	3	C	T	transition	A	A	synonymous	cytochrome c oxidase subunit I
KT361659	Amazona aestiva	COX1	CDS	5354	5983	6901	+	3	C	T	transition	L	L	synonymous	cytochrome c oxidase subunit I
KT361659	Amazona aestiva	COX1	CDS	5354	6070	6901	+	3	A	G	transition	G	G	synonymous	cytochrome c oxidase subunit I
KT361659	Amazona aestiva	COX1	CDS	5354	6172	6901	+	3	G	A	transition	M	M	synonymous	cytochrome c oxidase subunit I
KT361659	Amazona aestiva	COX1	CDS	5354	6175	6901	+	3	A	G	transition	V	V	synonymous	cytochrome c oxidase subunit I
KT361659	Amazona aestiva	COX1	CDS	5354	6208	6901	+	3	T	C	transition	F	F	synonymous	cytochrome c oxidase subunit I
KT361659	Amazona aestiva	COX1	CDS	5354	6223	6901	+	3	C	T	transition	H	H	synonymous	cytochrome c oxidase subunit I
KT361659	Amazona aestiva	COX1	CDS	5354	6238	6901	+	3	G	A	transition	V	V	synonymous	cytochrome c oxidase subunit I
KT361659	Amazona aestiva	COX1	CDS	5354	6256	6901	+	3	C	T	transition	T	T	synonymous	cytochrome c oxidase subunit I
KT361659	Amazona aestiva	COX1	CDS	5354	6406	6901	+	3	A	G	transition	G	G	synonymous	cytochrome c oxidase subunit I
KT361659	Amazona aestiva	COX1	CDS	5354	6424	6901	+	3	C	T	transition	V	V	synonymous	cytochrome c oxidase subunit I
KT361659	Amazona aestiva	COX1	CDS	5354	6440	6901	+	1	C	T	transition	L	L	synonymous	cytochrome c oxidase subunit I
KT361659	Amazona aestiva	COX1	CDS	5354	6493	6901	+	3	T	C	transition	V	V	synonymous	cytochrome c oxidase subunit I
KT361659	Amazona aestiva	COX1	CDS	5354	6688	6901	+	3	T	C	transition	D	D	synonymous	cytochrome c oxidase subunit I
KT361659	Amazona aestiva	COX1	CDS	5354	6811	6901	+	3	C	T	transition	S	S	synonymous	cytochrome c oxidase subunit I
KT361659	Amazona aestiva	COX1	CDS	5354	6823	6901	+	3	T	C	transition	T	T	synonymous	cytochrome c oxidase subunit I
KT361659	Amazona aestiva	COX1	CDS	5354	6850	6901	+	3	A	G	transition	P	P	synonymous	cytochrome c oxidase subunit I
KT361659	Amazona aestiva	COX1	CDS	5354	6892	6901	+	3	G	A	transition	V	V	synonymous	cytochrome c oxidase subunit I
KT361659	Amazona aestiva	tRNA-Asp	tRNA	6971	6999	7039	+		T	C	transition				transfer RNA aspartate
KT361659	Amazona aestiva	tRNA-Asp	tRNA	6971	7023	7039	+		G	A	transition				transfer RNA aspartate
KT361659	Amazona aestiva	COX2	CDS	7042	7052	7725	+	2	A	G	transition	H	R	missense	cytochrome c oxidase subunit II
KT361659	Amazona aestiva	COX2	CDS	7042	7119	7725	+	3	T	C	transition	H	H	synonymous	cytochrome c oxidase subunit II
KT361659	Amazona aestiva	COX2	CDS	7042	7122	7725	+	3	T	C	transition	A	A	synonymous	cytochrome c oxidase subunit II
KT361659	Amazona aestiva	COX2	CDS	7042	7131	7725	+	3	T	C	transition	V	V	synonymous	cytochrome c oxidase subunit II
KT361659	Amazona aestiva	COX2	CDS	7042	7134	7725	+	3	T	C	transition	A	A	synonymous	cytochrome c oxidase subunit II
KT361659	Amazona aestiva	COX2	CDS	7042	7257	7725	+	3	T	C	transition	L	L	synonymous	cytochrome c oxidase subunit II
KT361659	Amazona aestiva	COX2	CDS	7042	7260	7725	+	3	T	C	transition	I	I	synonymous	cytochrome c oxidase subunit II
KT361659	Amazona aestiva	COX2	CDS	7042	7425	7725	+	3	G	A	transition	P	P	synonymous	cytochrome c oxidase subunit II
KT361659	Amazona aestiva	COX2	CDS	7042	7484	7725	+	2	C	T	transition	P	L	missense	cytochrome c oxidase subunit II
KT361659	Amazona aestiva	COX2	CDS	7042	7530	7725	+	3	T	C	transition	A	A	synonymous	cytochrome c oxidase subunit II
KT361659	Amazona aestiva	COX2	CDS	7042	7539	7725	+	3	A	G	transition	T	T	synonymous	cytochrome c oxidase subunit II
KT361659	Amazona aestiva	COX2	CDS	7042	7566	7725	+	3	T	C	transition	P	P	synonymous	cytochrome c oxidase subunit II
KT361659	Amazona aestiva	COX2	CDS	7042	7623	7725	+	3	A	G	transition	Q	Q	synonymous	cytochrome c oxidase subunit II
KT361659	Amazona aestiva	COX2	CDS	7042	7662	7725	+	3	T	C	transition	P	P	synonymous	cytochrome c oxidase subunit II
KT361659	Amazona aestiva	COX2	CDS	7042	7717	7725	+	1	A	G	transition	M	V	missense	cytochrome c oxidase subunit II
KT361659	Amazona aestiva	tRNA-Lys	tRNA	7727	7779	7795	+		C	T	transition				transfer RNA lysine
KT361659	Amazona aestiva	ATP8	CDS	7797	7948	7964	+	2	T	C	transition	I	T	missense	ATP synthase F0 subunit 8
KT361659	Amazona aestiva	ATP6	CDS	7955	8009	8638	+	1	T	C	transition	L	L	synonymous	ATP synthase F0 subunit 6

Genbank ID	Organism	Gene	Type	Start	Actual Position	End	Strand	Codon Position	A. aestiva	A. barbadensis	Type of Mutation	A. aestiva AA	A. barbadensis AA	Type of Mutation	Product
KT361659	Amazona aestiva	ATP6	CDS	7955	8059	8638	+	3	T	C	transition	N	N	synonymous	ATP synthase F0 subunit 6
KT361659	Amazona aestiva	ATP6	CDS	7955	8069	8638	+	1	A	G	transition	I	V	missense	ATP synthase F0 subunit 6
KT361659	Amazona aestiva	ATP6	CDS	7955	8077	8638	+	3	T	C	transition	N	N	synonymous	ATP synthase F0 subunit 6
KT361659	Amazona aestiva	ATP6	CDS	7955	8107	8638	+	3	T	C	transition	T	T	synonymous	ATP synthase F0 subunit 6
KT361659	Amazona aestiva	ATP6	CDS	7955	8180	8638	+	1	T	C	transition	L	L	synonymous	ATP synthase F0 subunit 6
KT361659	Amazona aestiva	ATP6	CDS	7955	8195	8638	+	1	T	C	transition	L	L	synonymous	ATP synthase F0 subunit 6
KT361659	Amazona aestiva	ATP6	CDS	7955	8203	8638	+	3	T	C	transition	I	I	synonymous	ATP synthase F0 subunit 6
KT361659	Amazona aestiva	ATP6	CDS	7955	8212	8638	+	3	A	G	transition	L	L	synonymous	ATP synthase F0 subunit 6
KT361659	Amazona aestiva	ATP6	CDS	7955	8233	8638	+	3	C	T	transition	F	F	synonymous	ATP synthase F0 subunit 6
KT361659	Amazona aestiva	ATP6	CDS	7955	8236	8638	+	3	T	C	transition	T	T	synonymous	ATP synthase F0 subunit 6
KT361659	Amazona aestiva	ATP6	CDS	7955	8245	8638	+	3	T	C	transition	T	T	synonymous	ATP synthase F0 subunit 6
KT361659	Amazona aestiva	ATP6	CDS	7955	8413	8638	+	3	C	T	transition	R	R	synonymous	ATP synthase F0 subunit 6
KT361659	Amazona aestiva	ATP6	CDS	7955	8425	8638	+	3	G	T	transversion	L	L	synonymous	ATP synthase F0 subunit 6
KT361659	Amazona aestiva	ATP6	CDS	7955	8503	8638	+	3	C	T	transition	L	L	synonymous	ATP synthase F0 subunit 6
KT361659	Amazona aestiva	ATP6	CDS	7955	8521	8638	+	3	G	A	transition	V	V	synonymous	ATP synthase F0 subunit 6
KT361659	Amazona aestiva	ATP6	CDS	7955	8593	8638	+	3	T	C	transition	Y	Y	synonymous	ATP synthase F0 subunit 6
KT361659	Amazona aestiva	ATP6	CDS	7955	8617	8638	+	3	C	T	transition	L	L	synonymous	ATP synthase F0 subunit 6
KT361659	Amazona aestiva	COX3	CDS	8638	8673	9421	+	3	C	T	transition	D	D	synonymous	cytochrome c oxidase subunit III
KT361659	Amazona aestiva	COX3	CDS	8638	8676	9421	+	3	C	T	transition	P	P	synonymous	cytochrome c oxidase subunit III
KT361659	Amazona aestiva	COX3	CDS	8638	8697	9421	+	3	G	A	transition	G	G	synonymous	cytochrome c oxidase subunit III
KT361659	Amazona aestiva	COX3	CDS	8638	8715	9421	+	3	A	G	transition	L	L	synonymous	cytochrome c oxidase subunit III
KT361659	Amazona aestiva	COX3	CDS	8638	8728	9421	+	1	C	T	transition	L	L	synonymous	cytochrome c oxidase subunit III
KT361659	Amazona aestiva	COX3	CDS	8638	8790	9421	+	3	C	T	transition	I	I	synonymous	cytochrome c oxidase subunit III
KT361659	Amazona aestiva	COX3	CDS	8638	8850	9421	+	3	T	C	transition	H	H	synonymous	cytochrome c oxidase subunit III
KT361659	Amazona aestiva	COX3	CDS	8638	9090	9421	+	3	C	T	transition	I	I	synonymous	cytochrome c oxidase subunit III
KT361659	Amazona aestiva	COX3	CDS	8638	9104	9421	+	2	A	G	transition	Q	R	missense	cytochrome c oxidase subunit III
KT361659	Amazona aestiva	COX3	CDS	8638	9207	9421	+	3	C	T	transition	D	D	synonymous	cytochrome c oxidase subunit III
KT361659	Amazona aestiva	COX3	CDS	8638	9208	9421	+	1	A	G	transition	S	G	missense	cytochrome c oxidase subunit III
KT361659	Amazona aestiva	COX3	CDS	8638	9231	9421	+	3	C	T	transition	F	F	synonymous	cytochrome c oxidase subunit III
KT361659	Amazona aestiva	COX3	CDS	8638	9255	9421	+	3	C	T	transition	L	L	synonymous	cytochrome c oxidase subunit III
KT361659	Amazona aestiva	COX3	CDS	8638	9267	9421	+	3	T	C	transition	I	I	synonymous	cytochrome c oxidase subunit III
KT361659	Amazona aestiva	COX3	CDS	8638	9288	9421	+	3	C	T	transition	V	V	synonymous	cytochrome c oxidase subunit III
KT361659	Amazona aestiva	COX3	CDS	8638	9351	9421	+	3	A	T	transversion	A	A	synonymous	cytochrome c oxidase subunit III
KT361659	Amazona aestiva	COX3	CDS	8638	9366	9421	+	3	C	T	transition	H	H	synonymous	cytochrome c oxidase subunit III
KT361659	Amazona aestiva	COX3	CDS	8638	9390	9421	+	3	T	C	transition	F	F	synonymous	cytochrome c oxidase subunit III
KT361659	Amazona aestiva	ND3	CDS	9490	9522	9841	+	3	T	C	transition	T	T	synonymous	NADH dehydrogenase subunit 3
KT361659	Amazona aestiva	ND3	CDS	9490	9691	9841	+	3	C	T	transition	D	D	synonymous	NADH dehydrogenase subunit 3
KT361659	Amazona aestiva	ND3	CDS	9490	9751	9841	+	3	T	C	transition	T	T	synonymous	NADH dehydrogenase subunit 3
KT361659	Amazona aestiva	ND3	CDS	9490	9793	9841	+	3	G	A	transition	L	L	synonymous	NADH dehydrogenase subunit 3
KT361659	Amazona aestiva	tRNA-Arg	tRNA	9841	9891	9909	+		C	T	transition				transfer RNA arginine
KT361659	Amazona aestiva	ND4L	CDS	9911	9994	10207	+	3	C	T	transition	S	S	synonymous	NADH dehydrogenase subunit 4L

Genbank ID	Organism	Gene	Type	Start	Actual Position	End	Strand	Codon Position	A. aestiva	A. barbadensis	Type of Mutation	A. aestiva AA	A. barbadensis AA	Type of Mutation	Product
KT361659	Amazona aestiva	ND4L	CDS	9911	9998	10207	+	1	T	C	transition	L	L	synonymous	NADH dehydrogenase subunit 4L
KT361659	Amazona aestiva	ND4L	CDS	9911	10037	10207	+	1	G	A	transition	A	T	missense	NADH dehydrogenase subunit 4L
KT361659	Amazona aestiva	ND4L	CDS	9911	10054	10207	+	3	T	C	transition	P	P	synonymous	NADH dehydrogenase subunit 4L
KT361659	Amazona aestiva	ND4L	CDS	9911	10057	10207	+	3	C	T	transition	V	V	synonymous	NADH dehydrogenase subunit 4L
KT361659	Amazona aestiva	ND4L	CDS	9911	10111	10207	+	3	C	T	transition	S	S	synonymous	NADH dehydrogenase subunit 4L
KT361659	Amazona aestiva	ND4L	CDS	9911	10126	10207	+	3	C	T	transition	G	G	synonymous	NADH dehydrogenase subunit 4L
KT361659	Amazona aestiva	ND4L	CDS	9911	10175	10207	+	1	A	C	transversion	N	H	missense	NADH dehydrogenase subunit 4L
KT361659	Amazona aestiva	ND4	CDS	10201	10236	11593	+	3	T	C	transition	L	L	synonymous	NADH dehydrogenase subunit 4
KT361659	Amazona aestiva	ND4	CDS	10201	10286	11593	+	2	C	T	transition	T	M	missense	NADH dehydrogenase subunit 4
KT361659	Amazona aestiva	ND4	CDS	10201	10296	11593	+	3	G	A	transition	L	L	synonymous	NADH dehydrogenase subunit 4
KT361659	Amazona aestiva	ND4	CDS	10201	10450	11593	+	1	C	T	transition	L	L	synonymous	NADH dehydrogenase subunit 4
KT361659	Amazona aestiva	ND4	CDS	10201	10480	11593	+	1	A	G	transition	I	V	missense	NADH dehydrogenase subunit 4
KT361659	Amazona aestiva	ND4	CDS	10201	10497	11593	+	3	G	T	transversion	L	L	synonymous	NADH dehydrogenase subunit 4
KT361659	Amazona aestiva	ND4	CDS	10201	10659	11593	+	3	G	A	transition	T	T	synonymous	NADH dehydrogenase subunit 4
KT361659	Amazona aestiva	ND4	CDS	10201	10695	11593	+	3	G	A	transition	L	L	synonymous	NADH dehydrogenase subunit 4
KT361659	Amazona aestiva	ND4	CDS	10201	10827	11593	+	3	A	G	transition	V	V	synonymous	NADH dehydrogenase subunit 4
KT361659	Amazona aestiva	ND4	CDS	10201	10839	11593	+	3	G	A	transition	L	L	synonymous	NADH dehydrogenase subunit 4
KT361659	Amazona aestiva	ND4	CDS	10201	10924	11593	+	1	T	C	transition	L	L	synonymous	NADH dehydrogenase subunit 4
KT361659	Amazona aestiva	ND4	CDS	10201	10944	11593	+	3	G	A	transition	M	M	synonymous	NADH dehydrogenase subunit 4
KT361659	Amazona aestiva	ND4	CDS	10201	10947	11593	+	3	C	T	transition	R	R	synonymous	NADH dehydrogenase subunit 4
KT361659	Amazona aestiva	ND4	CDS	10201	11004	11593	+	3	T	C	transition	T	T	synonymous	NADH dehydrogenase subunit 4
KT361659	Amazona aestiva	ND4	CDS	10201	11007	11593	+	3	G	A	transition	L	L	synonymous	NADH dehydrogenase subunit 4
KT361659	Amazona aestiva	ND4	CDS	10201	11011	11593	+	1	T	C	transition	L	L	synonymous	NADH dehydrogenase subunit 4
KT361659	Amazona aestiva	ND4	CDS	10201	11016	11593	+	3	G	A	transition	W	W	synonymous	NADH dehydrogenase subunit 4
KT361659	Amazona aestiva	ND4	CDS	10201	11043	11593	+	3	T	C	transition	C	C	synonymous	NADH dehydrogenase subunit 4
KT361659	Amazona aestiva	ND4	CDS	10201	11112	11593	+	3	C	T	transition	A	A	synonymous	NADH dehydrogenase subunit 4
KT361659	Amazona aestiva	ND4	CDS	10201	11139	11593	+	3	T	C	transition	S	S	synonymous	NADH dehydrogenase subunit 4
KT361659	Amazona aestiva	ND4	CDS	10201	11172	11593	+	3	T	C	transition	H	H	synonymous	NADH dehydrogenase subunit 4
KT361659	Amazona aestiva	ND4	CDS	10201	11200	11593	+	1	C	T	transition	L	L	synonymous	NADH dehydrogenase subunit 4
KT361659	Amazona aestiva	ND4	CDS	10201	11262	11593	+	3	A	G	transition	Q	Q	synonymous	NADH dehydrogenase subunit 4
KT361659	Amazona aestiva	ND4	CDS	10201	11280	11593	+	3	G	A	transition	M	M	synonymous	NADH dehydrogenase subunit 4
KT361659	Amazona aestiva	ND4	CDS	10201	11382	11593	+	3	T	C	transition	S	S	synonymous	NADH dehydrogenase subunit 4
KT361659	Amazona aestiva	ND4	CDS	10201	11383	11593	+	1	A	G	transition	T	A	missense	NADH dehydrogenase subunit 4
KT361659	Amazona aestiva	ND4	CDS	10201	11410	11593	+	1	G	A	transition	A	T	missense	NADH dehydrogenase subunit 4
KT361659	Amazona aestiva	ND4	CDS	10201	11437	11593	+	1	C	T	transition	L	L	synonymous	NADH dehydrogenase subunit 4
KT361659	Amazona aestiva	ND4	CDS	10201	11517	11593	+	3	T	C	transition	H	H	synonymous	NADH dehydrogenase subunit 4
KT361659	Amazona aestiva	tRNA-His	tRNA	11594	11646	11662	+		G	A	transition				transfer RNA histidine
KT361659	Amazona aestiva	ND5	CDS	11799	11811	13622	+	1	T	C	transition	L	L	synonymous	NADH dehydrogenase subunit 5
KT361659	Amazona aestiva	ND5	CDS	11799	11858	13622	+	3	A	G	transition	T	T	synonymous	NADH dehydrogenase subunit 5
KT361659	Amazona aestiva	ND5	CDS	11799	11861	13622	+	3	C	T	transition	P	P	synonymous	NADH dehydrogenase subunit 5
KT361659	Amazona aestiva	ND5	CDS	11799	11903	13622	+	3	C	T	transition	P	P	synonymous	NADH dehydrogenase subunit 5

Genbank ID	Organism	Gene	Type	Start	Actual Position	End	Strand	Codon Position	A. aestiva	A. barbadensis	Type of Mutation	A. aestiva AA	A. barbadensis AA	Type of Mutation	Product
KT361659	Amazona aestiva	ND5	CDS	11799	11919	13622	+	1	A	G	transition	T	A	missense	NADH dehydrogenase subunit 5
KT361659	Amazona aestiva	ND5	CDS	11799	11930	13622	+	3	C	T	transition	T	T	synonymous	NADH dehydrogenase subunit 5
KT361659	Amazona aestiva	ND5	CDS	11799	11950	13622	+	2	C	T	transition	A	V	missense	NADH dehydrogenase subunit 5
KT361659	Amazona aestiva	ND5	CDS	11799	11972	13622	+	3	C	T	transition	S	S	synonymous	NADH dehydrogenase subunit 5
KT361659	Amazona aestiva	ND5	CDS	11799	12041	13622	+	3	C	T	transition	S	S	synonymous	NADH dehydrogenase subunit 5
KT361659	Amazona aestiva	ND5	CDS	11799	12095	13622	+	3	A	G	transition	T	T	synonymous	NADH dehydrogenase subunit 5
KT361659	Amazona aestiva	ND5	CDS	11799	12098	13622	+	3	G	A	transition	W	W	synonymous	NADH dehydrogenase subunit 5
KT361659	Amazona aestiva	ND5	CDS	11799	12143	13622	+	3	C	T	transition	H	H	synonymous	NADH dehydrogenase subunit 5
KT361659	Amazona aestiva	ND5	CDS	11799	12144	13622	+	1	G	A	transition	A	T	missense	NADH dehydrogenase subunit 5
KT361659	Amazona aestiva	ND5	CDS	11799	12287	13622	+	3	A	G	transition	R	R	synonymous	NADH dehydrogenase subunit 5
KT361659	Amazona aestiva	ND5	CDS	11799	12323	13622	+	3	T	C	transition	L	L	synonymous	NADH dehydrogenase subunit 5
KT361659	Amazona aestiva	ND5	CDS	11799	12424	13622	+	2	C	T	transition	T	I	missense	NADH dehydrogenase subunit 5
KT361659	Amazona aestiva	ND5	CDS	11799	12449	13622	+	3	T	C	transition	L	L	synonymous	NADH dehydrogenase subunit 5
KT361659	Amazona aestiva	ND5	CDS	11799	12461	13622	+	3	T	C	transition	A	A	synonymous	NADH dehydrogenase subunit 5
KT361659	Amazona aestiva	ND5	CDS	11799	12494	13622	+	3	C	T	transition	P	P	synonymous	NADH dehydrogenase subunit 5
KT361659	Amazona aestiva	ND5	CDS	11799	12506	13622	+	3	T	C	transition	A	A	synonymous	NADH dehydrogenase subunit 5
KT361659	Amazona aestiva	ND5	CDS	11799	12515	13622	+	3	A	G	transition	E	E	synonymous	NADH dehydrogenase subunit 5
KT361659	Amazona aestiva	ND5	CDS	11799	12533	13622	+	3	T	C	transition	S	S	synonymous	NADH dehydrogenase subunit 5
KT361659	Amazona aestiva	ND5	CDS	11799	12551	13622	+	3	C	T	transition	S	S	synonymous	NADH dehydrogenase subunit 5
KT361659	Amazona aestiva	ND5	CDS	11799	12584	13622	+	3	T	C	transition	I	I	synonymous	NADH dehydrogenase subunit 5
KT361659	Amazona aestiva	ND5	CDS	11799	12695	13622	+	3	C	T	transition	I	I	synonymous	NADH dehydrogenase subunit 5
KT361659	Amazona aestiva	ND5	CDS	11799	12833	13622	+	3	G	A	transition	G	G	synonymous	NADH dehydrogenase subunit 5
KT361659	Amazona aestiva	ND5	CDS	11799	12866	13622	+	3	T	C	transition	D	D	synonymous	NADH dehydrogenase subunit 5
KT361659	Amazona aestiva	ND5	CDS	11799	12895	13622	+	2	C	T	transition	T	M	missense	NADH dehydrogenase subunit 5
KT361659	Amazona aestiva	ND5	CDS	11799	12947	13622	+	3	G	A	transition	G	G	synonymous	NADH dehydrogenase subunit 5
KT361659	Amazona aestiva	ND5	CDS	11799	13004	13622	+	3	T	C	transition	T	T	synonymous	NADH dehydrogenase subunit 5
KT361659	Amazona aestiva	ND5	CDS	11799	13029	13622	+	1	A	G	transition	T	A	missense	NADH dehydrogenase subunit 5
KT361659	Amazona aestiva	ND5	CDS	11799	13122	13622	+	1	G	A	transition	V	I	missense	NADH dehydrogenase subunit 5
KT361659	Amazona aestiva	ND5	CDS	11799	13127	13622	+	3	A	G	transition	P	P	synonymous	NADH dehydrogenase subunit 5
KT361659	Amazona aestiva	ND5	CDS	11799	13172	13622	+	3	A	G	transition	L	L	synonymous	NADH dehydrogenase subunit 5
KT361659	Amazona aestiva	ND5	CDS	11799	13176	13622	+	1	T	C	transition	L	L	synonymous	NADH dehydrogenase subunit 5
KT361659	Amazona aestiva	ND5	CDS	11799	13211	13622	+	3	G	A	transition	S	S	synonymous	NADH dehydrogenase subunit 5
KT361659	Amazona aestiva	ND5	CDS	11799	13217	13622	+	3	A	G	transition	M	M	synonymous	NADH dehydrogenase subunit 5
KT361659	Amazona aestiva	ND5	CDS	11799	13220	13622	+	3	C	T	transition	T	T	synonymous	NADH dehydrogenase subunit 5
KT361659	Amazona aestiva	ND5	CDS	11799	13223	13622	+	3	A	G	transition	P	P	synonymous	NADH dehydrogenase subunit 5
KT361659	Amazona aestiva	ND5	CDS	11799	13274	13622	+	3	C	T	transition	I	I	synonymous	NADH dehydrogenase subunit 5
KT361659	Amazona aestiva	ND5	CDS	11799	13292	13622	+	3	A	G	transition	G	G	synonymous	NADH dehydrogenase subunit 5
KT361659	Amazona aestiva	ND5	CDS	11799	13299	13622	+	1	C	T	transition	L	L	synonymous	NADH dehydrogenase subunit 5
KT361659	Amazona aestiva	ND5	CDS	11799	13377	13622	+	1	T	C	transition	L	L	synonymous	NADH dehydrogenase subunit 5
KT361659	Amazona aestiva	ND5	CDS	11799	13398	13622	+	1	G	A	transition	A	T	missense	NADH dehydrogenase subunit 5
KT361659	Amazona aestiva	ND5	CDS	11799	13444	13622	+	2	C	T	transition	T	I	missense	NADH dehydrogenase subunit 5

Genbank ID	Organism	Gene	Type	Start	Actual Position	End	Strand	Codon Position	A. aestiva	A. barbadensis	Type of Mutation	A. aestiva AA	A. barbadensis AA	Type of Mutation	Product
KT361659	Amazona aestiva	ND5	CDS	11799	13448	13622	+	3	T	C	transition	A	A	synonymous	NADH dehydrogenase subunit 5
KT361659	Amazona aestiva	ND5	CDS	11799	13460	13622	+	3	A	G	transition	M	M	synonymous	NADH dehydrogenase subunit 5
KT361659	Amazona aestiva	ND5	CDS	11799	13484	13622	+	3	C	T	transition	I	I	synonymous	NADH dehydrogenase subunit 5
KT361659	Amazona aestiva	ND5	CDS	11799	13499	13622	+	3	C	T	transition	L	L	synonymous	NADH dehydrogenase subunit 5
KT361659	Amazona aestiva	ND5	CDS	11799	13505	13622	+	3	T	C	transition	N	N	synonymous	NADH dehydrogenase subunit 5
KT361659	Amazona aestiva	ND5	CDS	11799	13517	13622	+	3	G	A	transition	M	M	synonymous	NADH dehydrogenase subunit 5
KT361659	Amazona aestiva	ND5	CDS	11799	13538	13622	+	3	G	A	transition	T	T	synonymous	NADH dehydrogenase subunit 5
KT361659	Amazona aestiva	ND5	CDS	11799	13547	13622	+	3	G	A	transition	K	K	synonymous	NADH dehydrogenase subunit 5
KT361659	Amazona aestiva	ND5	CDS	11799	13602	13622	+	1	T	C	transition	L	L	synonymous	NADH dehydrogenase subunit 5
KT361659	Amazona aestiva	ND5	CDS	11799	13605	13622	+	1	A	T	transversion	M	L	missense	NADH dehydrogenase subunit 5
KT361659	Amazona aestiva	ND5	CDS	11799	13607	13622	+	3	.	C	INDEL		L -> Coded by the codon with insertions	missense	NADH dehydrogenase subunit 5
KT361659	Amazona aestiva	ND5	CDS	11799	13607	13622	+	3	.	T	INDEL		L -> Coded by the codon with insertions	missense	NADH dehydrogenase subunit 5
KT361659	Amazona aestiva	ND5	CDS	11799	13611	13622	+	1	.	C	INDEL		I -> Coded by the codon with insertions	missense	NADH dehydrogenase subunit 5
KT361659	Amazona aestiva	ND5	CDS	11799	13618	13622	+	2	C	T	transition	T	I	missense	NADH dehydrogenase subunit 5
KT361659	Amazona aestiva	CYTB	CDS	13622	13673	14761	+	1	T	C	transition	S	P	missense	cytochrome b
KT361659	Amazona aestiva	CYTB	CDS	13622	13687	14761	+	3	A	G	transition	L	L	synonymous	cytochrome b
KT361659	Amazona aestiva	CYTB	CDS	13622	13693	14761	+	3	G	A	transition	T	T	synonymous	cytochrome b
KT361659	Amazona aestiva	CYTB	CDS	13622	13768	14761	+	3	T	C	transition	G	G	synonymous	cytochrome b
KT361659	Amazona aestiva	CYTB	CDS	13622	13798	14761	+	3	C	T	transition	D	D	synonymous	cytochrome b
KT361659	Amazona aestiva	CYTB	CDS	13622	13807	14761	+	3	A	G	transition	L	L	synonymous	cytochrome b
KT361659	Amazona aestiva	CYTB	CDS	13622	13822	14761	+	3	G	A	transition	V	V	synonymous	cytochrome b
KT361659	Amazona aestiva	CYTB	CDS	13622	13834	14761	+	3	C	T	transition	C	C	synonymous	cytochrome b
KT361659	Amazona aestiva	CYTB	CDS	13622	13852	14761	+	3	C	T	transition	G	G	synonymous	cytochrome b
KT361659	Amazona aestiva	CYTB	CDS	13622	13873	14761	+	3	C	T	transition	H	H	synonymous	cytochrome b
KT361659	Amazona aestiva	CYTB	CDS	13622	13936	14761	+	3	T	C	transition	Y	Y	synonymous	cytochrome b
KT361659	Amazona aestiva	CYTB	CDS	13622	13948	14761	+	3	G	A	transition	L	L	synonymous	cytochrome b
KT361659	Amazona aestiva	CYTB	CDS	13622	14038	14761	+	3	A	G	transition	M	M	synonymous	cytochrome b
KT361659	Amazona aestiva	CYTB	CDS	13622	14053	14761	+	3	C	T	transition	A	A	synonymous	cytochrome b
KT361659	Amazona aestiva	CYTB	CDS	13622	14077	14761	+	3	T	C	transition	S	S	synonymous	cytochrome b
KT361659	Amazona aestiva	CYTB	CDS	13622	14080	14761	+	3	T	C	transition	A	A	synonymous	cytochrome b
KT361659	Amazona aestiva	CYTB	CDS	13622	14098	14761	+	3	G	A	transition	Q	Q	synonymous	cytochrome b
KT361659	Amazona aestiva	CYTB	CDS	13622	14149	14761	+	3	G	A	transition	L	L	synonymous	cytochrome b
KT361659	Amazona aestiva	CYTB	CDS	13622	14155	14761	+	3	A	G	transition	R	R	synonymous	cytochrome b
KT361659	Amazona aestiva	CYTB	CDS	13622	14164	14761	+	3	C	T	transition	A	A	synonymous	cytochrome b
KT361659	Amazona aestiva	CYTB	CDS	13622	14222	14761	+	1	T	C	transition	L	L	synonymous	cytochrome b
KT361659	Amazona aestiva	CYTB	CDS	13622	14248	14761	+	3	T	C	transition	P	P	synonymous	cytochrome b
KT361659	Amazona aestiva	CYTB	CDS	13622	14320	14761	+	3	C	T	transition	F	F	synonymous	cytochrome b
KT361659	Amazona aestiva	CYTB	CDS	13622	14344	14761	+	3	C	T	transition	S	S	synonymous	cytochrome b
KT361659	Amazona aestiva	CYTB	CDS	13622	14345	14761	+	1	A	G	transition	T	A	missense	cytochrome b

Genbank ID	Organism	Gene	Type	Start	Actual Position	End	Strand	Codon Position	A. aestiva	A. barbadensis	Type of Mutation	A. aestiva AA	A. barbadensis AA	Type of Mutation	Product
KT361659	Amazona aestiva	CYTB	CDS	13622	14416	14761	+	3	C	T	transition	T	T	synonymous	cytochrome b
KT361659	Amazona aestiva	CYTB	CDS	13622	14458	14761	+	3	C	T	transition	Y	Y	synonymous	cytochrome b
KT361659	Amazona aestiva	CYTB	CDS	13622	14491	14761	+	3	G	A	transition	G	G	synonymous	cytochrome b
KT361659	Amazona aestiva	CYTB	CDS	13622	14524	14761	+	3	C	T	transition	I	I	synonymous	cytochrome b
KT361659	Amazona aestiva	CYTB	CDS	13622	14557	14761	+	3	A	G	transition	K	K	synonymous	cytochrome b
KT361659	Amazona aestiva	CYTB	CDS	13622	14590	14761	+	3	A	G	transition	Q	Q	synonymous	cytochrome b
KT361659	Amazona aestiva	CYTB	CDS	13622	14606	14761	+	1	T	C	transition	L	L	synonymous	cytochrome b
KT361659	Amazona aestiva	CYTB	CDS	13622	14624	14761	+	1	A	G	transition	I	V	missense	cytochrome b
KT361659	Amazona aestiva	CYTB	CDS	13622	14635	14761	+	3	A	G	transition	W	W	synonymous	cytochrome b
KT361659	Amazona aestiva	CYTB	CDS	13622	14641	14761	+	3	G	A	transition	G	G	synonymous	cytochrome b
KT361659	Amazona aestiva	CYTB	CDS	13622	14671	14761	+	3	T	C	transition	T	T	synonymous	cytochrome b
KT361659	Amazona aestiva	CYTB	CDS	13622	14677	14761	+	3	G	A	transition	G	G	synonymous	cytochrome b
KT361659	Amazona aestiva	CYTB	CDS	13622	14683	14761	+	3	G	A	transition	L	L	synonymous	cytochrome b
KT361659	Amazona aestiva	CYTB	CDS	13622	14693	14761	+	1	G	A	transition	A	T	missense	cytochrome b
KT361659	Amazona aestiva	CYTB	CDS	13622	14704	14761	+	3	T	C	transition	S	S	synonymous	cytochrome b
KT361659	Amazona aestiva	pseudoND6	pseudogene	14831	14837	14890	+		C	T	transition				pseudoND6
KT361659	Amazona aestiva	pseudoND6	pseudogene	14831	14866	14890	+		.	C	INDEL				pseudoND6
KT361659	Amazona aestiva	pseudo-tRNA	pseudogene	14891	14930	14956	+		G	A	transition				pseudo-tRNA-Glu
KT361659	Amazona aestiva	pseudo-tRNA	pseudogene	14891	14936	14956	+		.	A	INDEL				pseudo-tRNA-Glu
KT361659	Amazona aestiva	pseudo-tRNA	pseudogene	14891	14946	14956	+		T	C	transition				pseudo-tRNA-Glu
KT361659	Amazona aestiva	ControlRegion1	Control Region	14957	14959	16521	+		C	.	INDEL				Control Region 1
KT361659	Amazona aestiva	ControlRegion1	Control Region	14957	14960	16521	+		C	.	INDEL				Control Region 1
KT361659	Amazona aestiva	ControlRegion1	Control Region	14957	14961	16521	+		C	.	INDEL				Control Region 1
KT361659	Amazona aestiva	ControlRegion1	Control Region	14957	14985	16521	+		.	C	INDEL				Control Region 1
KT361659	Amazona aestiva	ControlRegion1	Control Region	14957	14992	16521	+		T	C	transition				Control Region 1
KT361659	Amazona aestiva	ControlRegion1	Control Region	14957	15007	16521	+		T	C	transition				Control Region 1
KT361659	Amazona aestiva	ControlRegion1	Control Region	14957	15023	16521	+		A	G	transition				Control Region 1
KT361659	Amazona aestiva	ControlRegion1	Control Region	14957	15024	16521	+		G	A	transition				Control Region 1
KT361659	Amazona aestiva	ControlRegion1	Control Region	14957	15033	16521	+		G	A	transition				Control Region 1
KT361659	Amazona aestiva	ControlRegion1	Control Region	14957	15034	16521	+		T	C	transition				Control Region 1
KT361659	Amazona aestiva	ControlRegion1	Control Region	14957	15036	16521	+		T	G	transversion				Control Region 1
KT361659	Amazona aestiva	ControlRegion1	Control Region	14957	15046	16521	+		C	T	transition				Control Region 1
KT361659	Amazona aestiva	ControlRegion1	Control Region	14957	15051	16521	+		T	C	transition				Control Region 1
KT361659	Amazona aestiva	ControlRegion1	Control Region	14957	15052	16521	+		T	C	transition				Control Region 1
KT361659	Amazona aestiva	ControlRegion1	Control Region	14957	15055	16521	+		T	C	transition				Control Region 1
KT361659	Amazona aestiva	ControlRegion1	Control Region	14957	15061	16521	+		A	G	transition				Control Region 1
KT361659	Amazona aestiva	ControlRegion1	Control Region	14957	15064	16521	+		G	A	transition				Control Region 1
KT361659	Amazona aestiva	ControlRegion1	Control Region	14957	15066	16521	+		C	T	transition				Control Region 1
KT361659	Amazona aestiva	ControlRegion1	Control Region	14957	15082	16521	+		T	C	transition				Control Region 1
KT361659	Amazona aestiva	ControlRegion1	Control Region	14957	15101	16521	+		T	C	transition				Control Region 1
KT361659	Amazona aestiva	ControlRegion1	Control Region	14957	15111	16521	+		C	T	transition				Control Region 1

Genbank ID	Organism	Gene	Type	Start	Actual Position	End	Strand	Codon Position	A. aestiva	A. barbadensis	Type of Mutation	A. aestiva AA	A. barbadensis AA	Type of Mutation	Product
KT361659	Amazona aestiva	ControlRegion1	Control Region	14957	15121	16521	+		T	C	transition				Control Region 1
KT361659	Amazona aestiva	ControlRegion1	Control Region	14957	15126	16521	+		C	T	transition				Control Region 1
KT361659	Amazona aestiva	ControlRegion1	Control Region	14957	15143	16521	+		G	A	transition				Control Region 1
KT361659	Amazona aestiva	ControlRegion1	Control Region	14957	15153	16521	+		G	A	transition				Control Region 1
KT361659	Amazona aestiva	ControlRegion1	Control Region	14957	15156	16521	+		C	T	transition				Control Region 1
KT361659	Amazona aestiva	ControlRegion1	Control Region	14957	15158	16521	+		T	C	transition				Control Region 1
KT361659	Amazona aestiva	ControlRegion1	Control Region	14957	15165	16521	+		C	T	transition				Control Region 1
KT361659	Amazona aestiva	ControlRegion1	Control Region	14957	15168	16521	+		A	G	transition				Control Region 1
KT361659	Amazona aestiva	ControlRegion1	Control Region	14957	15169	16521	+		T	C	transition				Control Region 1
KT361659	Amazona aestiva	ControlRegion1	Control Region	14957	15182	16521	+		A	G	transition				Control Region 1
KT361659	Amazona aestiva	ControlRegion1	Control Region	14957	15185	16521	+		C	T	transition				Control Region 1
KT361659	Amazona aestiva	ControlRegion1	Control Region	14957	15214	16521	+		T	C	transition				Control Region 1
KT361659	Amazona aestiva	ControlRegion1	Control Region	14957	15216	16521	+		C	T	transition				Control Region 1
KT361659	Amazona aestiva	ControlRegion1	Control Region	14957	15234	16521	+		T	C	transition				Control Region 1
KT361659	Amazona aestiva	ControlRegion1	Control Region	14957	15235	16521	+		T	C	transition				Control Region 1
KT361659	Amazona aestiva	ControlRegion1	Control Region	14957	15238	16521	+		C	T	transition				Control Region 1
KT361659	Amazona aestiva	ControlRegion1	Control Region	14957	15245	16521	+		G	A	transition				Control Region 1
KT361659	Amazona aestiva	ControlRegion1	Control Region	14957	15246	16521	+		A	G	transition				Control Region 1
KT361659	Amazona aestiva	ControlRegion1	Control Region	14957	15248	16521	+		G	C	transversion				Control Region 1
KT361659	Amazona aestiva	ControlRegion1	Control Region	14957	15253	16521	+		C	T	transition				Control Region 1
KT361659	Amazona aestiva	ControlRegion1	Control Region	14957	15257	16521	+		C	T	transition				Control Region 1
KT361659	Amazona aestiva	ControlRegion1	Control Region	14957	15258	16521	+		T	C	transition				Control Region 1
KT361659	Amazona aestiva	ControlRegion1	Control Region	14957	15260	16521	+		T	A	transversion				Control Region 1
KT361659	Amazona aestiva	ControlRegion1	Control Region	14957	15265	16521	+		T	A	transversion				Control Region 1
KT361659	Amazona aestiva	ControlRegion1	Control Region	14957	15276	16521	+		T	C	transition				Control Region 1
KT361659	Amazona aestiva	ControlRegion1	Control Region	14957	15293	16521	+		T	C	transition				Control Region 1
KT361659	Amazona aestiva	ControlRegion1	Control Region	14957	15686	16521	+		A	G	transition				Control Region 1
KT361659	Amazona aestiva	ControlRegion1	Control Region	14957	15834	16521	+		C	G	transversion				Control Region 1
KT361659	Amazona aestiva	ControlRegion1	Control Region	14957	15838	16521	+		C	T	transition				Control Region 1
KT361659	Amazona aestiva	ControlRegion1	Control Region	14957	15916	16521	+		A	G	transition				Control Region 1
KT361659	Amazona aestiva	ControlRegion1	Control Region	14957	15945	16521	+		C	T	transition				Control Region 1
KT361659	Amazona aestiva	ControlRegion1	Control Region	14957	15946	16521	+		A	G	transition				Control Region 1
KT361659	Amazona aestiva	ControlRegion1	Control Region	14957	15954	16521	+		C	T	transition				Control Region 1
KT361659	Amazona aestiva	ControlRegion1	Control Region	14957	15955	16521	+		G	T	transversion				Control Region 1
KT361659	Amazona aestiva	ControlRegion1	Control Region	14957	15957	16521	+		A	G	transition				Control Region 1
KT361659	Amazona aestiva	ControlRegion1	Control Region	14957	15981	16521	+		C	T	transition				Control Region 1
KT361659	Amazona aestiva	ControlRegion1	Control Region	14957	16065	16521	+		C	T	transition				Control Region 1
KT361659	Amazona aestiva	ControlRegion1	Control Region	14957	16230	16521	+		.	T	INDEL				Control Region 1
KT361659	Amazona aestiva	ControlRegion1	Control Region	14957	16276	16521	+		.	T	INDEL				Control Region 1
KT361659	Amazona aestiva	ControlRegion1	Control Region	14957	16276	16521	+		.	T	INDEL				Control Region 1
KT361659	Amazona aestiva	ControlRegion1	Control Region	14957	16276	16521	+		.	C	INDEL				Control Region 1

Genbank ID	Organism	Gene	Type	Start	Actual Position	End	Strand	Codon Position	A. aestiva	A. barbadensis	Type of Mutation	A. aestiva AA	A. barbadensis AA	Type of Mutation	Product
KT361659	Amazona aestiva	ControlRegion1	Control Region	14957	16276	16521	+		.	T	INDEL				Control Region 1
KT361659	Amazona aestiva	ControlRegion1	Control Region	14957	16276	16521	+		.	T	INDEL				Control Region 1
KT361659	Amazona aestiva	ControlRegion1	Control Region	14957	16276	16521	+		.	T	INDEL				Control Region 1
KT361659	Amazona aestiva	ControlRegion1	Control Region	14957	16276	16521	+		.	G	INDEL				Control Region 1
KT361659	Amazona aestiva	ControlRegion1	Control Region	14957	16280	16521	+		.	T	INDEL				Control Region 1
KT361659	Amazona aestiva	ControlRegion1	Control Region	14957	16280	16521	+		.	C	INDEL				Control Region 1
KT361659	Amazona aestiva	ControlRegion1	Control Region	14957	16281	16521	+		.	T	INDEL				Control Region 1
KT361659	Amazona aestiva	ControlRegion1	Control Region	14957	16337	16521	+		.	T	INDEL				Control Region 1
KT361659	Amazona aestiva	ControlRegion1	Control Region	14957	16337	16521	+		.	T	INDEL				Control Region 1
KT361659	Amazona aestiva	ControlRegion1	Control Region	14957	16337	16521	+		.	T	INDEL				Control Region 1
KT361659	Amazona aestiva	ControlRegion1	Control Region	14957	16338	16521	+		.	G	INDEL				Control Region 1
KT361659	Amazona aestiva	ControlRegion1	Control Region	14957	16338	16521	+		.	T	INDEL				Control Region 1
KT361659	Amazona aestiva	ControlRegion1	Control Region	14957	16338	16521	+		.	T	INDEL				Control Region 1
KT361659	Amazona aestiva	ControlRegion1	Control Region	14957	16338	16521	+		.	G	INDEL				Control Region 1
KT361659	Amazona aestiva	ControlRegion1	Control Region	14957	16341	16521	+		.	C	INDEL				Control Region 1
KT361659	Amazona aestiva	ControlRegion1	Control Region	14957	16342	16521	+		A	G	transition				Control Region 1
KT361659	Amazona aestiva	ControlRegion1	Control Region	14957	16345	16521	+		.	T	INDEL				Control Region 1
KT361659	Amazona aestiva	ControlRegion1	Control Region	14957	16345	16521	+		.	T	INDEL				Control Region 1
KT361659	Amazona aestiva	ControlRegion1	Control Region	14957	16347	16521	+		.	G	INDEL				Control Region 1
KT361659	Amazona aestiva	ControlRegion1	Control Region	14957	16352	16521	+		.	T	INDEL				Control Region 1
KT361659	Amazona aestiva	ControlRegion1	Control Region	14957	16352	16521	+		.	C	INDEL				Control Region 1
KT361659	Amazona aestiva	ControlRegion1	Control Region	14957	16352	16521	+		.	G	INDEL				Control Region 1
KT361659	Amazona aestiva	ControlRegion1	Control Region	14957	16352	16521	+		.	C	INDEL				Control Region 1
KT361659	Amazona aestiva	ControlRegion1	Control Region	14957	16352	16521	+		.	T	INDEL				Control Region 1
KT361659	Amazona aestiva	ControlRegion1	Control Region	14957	16352	16521	+		.	G	INDEL				Control Region 1
KT361659	Amazona aestiva	ControlRegion1	Control Region	14957	16352	16521	+		.	T	INDEL				Control Region 1
KT361659	Amazona aestiva	ControlRegion1	Control Region	14957	16352	16521	+		.	T	INDEL				Control Region 1
KT361659	Amazona aestiva	ControlRegion1	Control Region	14957	16352	16521	+		.	T	INDEL				Control Region 1
KT361659	Amazona aestiva	ControlRegion1	Control Region	14957	16352	16521	+		.	C	INDEL				Control Region 1
KT361659	Amazona aestiva	ControlRegion1	Control Region	14957	16352	16521	+		.	G	INDEL				Control Region 1
KT361659	Amazona aestiva	ControlRegion1	Control Region	14957	16352	16521	+		.	T	INDEL				Control Region 1
KT361659	Amazona aestiva	ControlRegion1	Control Region	14957	16352	16521	+		.	C	INDEL				Control Region 1
KT361659	Amazona aestiva	ControlRegion1	Control Region	14957	16358	16521	+		.	T	INDEL				Control Region 1
KT361659	Amazona aestiva	ControlRegion1	Control Region	14957	16358	16521	+		.	T	INDEL				Control Region 1
KT361659	Amazona aestiva	ControlRegion1	Control Region	14957	16358	16521	+		.	A	INDEL				Control Region 1
KT361659	Amazona aestiva	ControlRegion1	Control Region	14957	16360	16521	+		.	T	INDEL				Control Region 1
KT361659	Amazona aestiva	ControlRegion1	Control Region	14957	16360	16521	+		.	T	INDEL				Control Region 1
KT361659	Amazona aestiva	ControlRegion1	Control Region	14957	16360	16521	+		.	G	INDEL				Control Region 1
KT361659	Amazona aestiva	ControlRegion1	Control Region	14957	16363	16521	+		.	T	INDEL				Control Region 1
KT361659	Amazona aestiva	ControlRegion1	Control Region	14957	16363	16521	+		.	C	INDEL				Control Region 1
KT361659	Amazona aestiva	ControlRegion1	Control Region	14957	16363	16521	+		.	T	INDEL				Control Region 1

Genbank ID	Organism	Gene	Type	Start	Actual Position	End	Strand	Codon Position	A. aestiva	A. barbadensis	Type of Mutation	A. aestiva AA	A. barbadensis AA	Type of Mutation	Product
KT361659	Amazona aestiva	ControlRegion1	Control Region	14957	16363	16521	+		.	T	INDEL				Control Region 1
KT361659	Amazona aestiva	ControlRegion1	Control Region	14957	16363	16521	+		.	G	INDEL				Control Region 1
KT361659	Amazona aestiva	ControlRegion1	Control Region	14957	16363	16521	+		.	C	INDEL				Control Region 1
KT361659	Amazona aestiva	ControlRegion1	Control Region	14957	16363	16521	+		.	C	INDEL				Control Region 1
KT361659	Amazona aestiva	ControlRegion1	Control Region	14957	16368	16521	+		.	G	INDEL				Control Region 1
KT361659	Amazona aestiva	ControlRegion1	Control Region	14957	16368	16521	+		.	C	INDEL				Control Region 1
KT361659	Amazona aestiva	ControlRegion1	Control Region	14957	16368	16521	+		.	T	INDEL				Control Region 1
KT361659	Amazona aestiva	ControlRegion1	Control Region	14957	16368	16521	+		.	T	INDEL				Control Region 1
KT361659	Amazona aestiva	ControlRegion1	Control Region	14957	16368	16521	+		.	T	INDEL				Control Region 1
KT361659	Amazona aestiva	ControlRegion1	Control Region	14957	16368	16521	+		.	A	INDEL				Control Region 1
KT361659	Amazona aestiva	ControlRegion1	Control Region	14957	16372	16521	+		.	T	INDEL				Control Region 1
KT361659	Amazona aestiva	ControlRegion1	Control Region	14957	16372	16521	+		.	A	INDEL				Control Region 1
KT361659	Amazona aestiva	ControlRegion1	Control Region	14957	16372	16521	+		.	C	INDEL				Control Region 1
KT361659	Amazona aestiva	ControlRegion1	Control Region	14957	16372	16521	+		.	C	INDEL				Control Region 1
KT361659	Amazona aestiva	ControlRegion1	Control Region	14957	16372	16521	+		.	C	INDEL				Control Region 1
KT361659	Amazona aestiva	ControlRegion1	Control Region	14957	16377	16521	+		T	C	transition				Control Region 1
KT361659	Amazona aestiva	ControlRegion1	Control Region	14957	16382	16521	+		T	.	INDEL				Control Region 1
KT361659	Amazona aestiva	ControlRegion1	Control Region	14957	16383	16521	+		T	C	transition				Control Region 1
KT361659	Amazona aestiva	ControlRegion1	Control Region	14957	16396	16521	+		T	C	transition				Control Region 1
KT361659	Amazona aestiva	ControlRegion1	Control Region	14957	16397	16521	+		C	T	transition				Control Region 1
KT361659	Amazona aestiva	ControlRegion1	Control Region	14957	16401	16521	+		T	C	transition				Control Region 1
KT361659	Amazona aestiva	ControlRegion1	Control Region	14957	16412	16521	+		A	T	transversion				Control Region 1
KT361659	Amazona aestiva	ControlRegion1	Control Region	14957	16436	16521	+		C	T	transition				Control Region 1
KT361659	Amazona aestiva	ControlRegion1	Control Region	14957	16438	16521	+		T	C	transition				Control Region 1
KT361659	Amazona aestiva	ControlRegion1	Control Region	14957	16443	16521	+		T	C	transition				Control Region 1
KT361659	Amazona aestiva	ControlRegion1	Control Region	14957	16446	16521	+		G	C	transversion				Control Region 1
KT361659	Amazona aestiva	ControlRegion1	Control Region	14957	16447	16521	+		C	T	transition				Control Region 1
KT361659	Amazona aestiva	ControlRegion1	Control Region	14957	16451	16521	+		T	G	transversion				Control Region 1
KT361659	Amazona aestiva	ControlRegion1	Control Region	14957	16468	16521	+		.	T	INDEL				Control Region 1
KT361659	Amazona aestiva	ControlRegion1	Control Region	14957	16475	16521	+		T	C	transition				Control Region 1
KT361659	Amazona aestiva	ControlRegion1	Control Region	14957	16477	16521	+		A	G	transition				Control Region 1
KT361659	Amazona aestiva	ControlRegion1	Control Region	14957	16496	16521	+		T	.	INDEL				Control Region 1
KT361659	Amazona aestiva	ControlRegion1	Control Region	14957	16498	16521	+		C	G	transversion				Control Region 1
KT361659	Amazona aestiva	ControlRegion1	Control Region	14957	16510	16521	+		.	A	INDEL				Control Region 1
KT361659	Amazona aestiva	ControlRegion1	Control Region	14957	16518	16521	+		C	.	INDEL				Control Region 1
KT361659	Amazona aestiva	ControlRegion1	Control Region	14957	16519	16521	+		C	.	INDEL				Control Region 1
KT361659	Amazona aestiva	tRNA-Pro	tRNA	16522	16545	16590	-		G	A	transition				transfer RNA proline
KT361659	Amazona aestiva	Between tRNA-Pro and ND6	INTERGENIC	NULL	16592	NULL	NULL	NULL	G	A	transition	NULL	NULL	intergenic	NULL
KT361659	Amazona aestiva	ND6	CDS	16594	16621	17112	-	3	G	T	transversion	L	L	synonymous	NADH dehydrogenase subunit 6
KT361659	Amazona aestiva	ND6	CDS	16594	16672	17112	-	3	T	C	transition	G	G	synonymous	NADH dehydrogenase subunit 6

Genbank ID	Organism	Gene	Type	Start	Actual Position	End	Strand	Codon Position	A. aestiva	A. barbadensis	Type of Mutation	A. aestiva AA	A. barbadensis AA	Type of Mutation	Product
KT361659	Amazona aestiva	ND6	CDS	16594	16748	17112	-	2	C	T	transition	A	M	missense	NADH dehydrogenase subunit 6
KT361659	Amazona aestiva	ND6	CDS	16594	16749	17112	-	1	G	A	transition	A	M	missense	NADH dehydrogenase subunit 6
KT361659	Amazona aestiva	ND6	CDS	16594	16769	17112	-	2	G	A	transition	G	E	missense	NADH dehydrogenase subunit 6
KT361659	Amazona aestiva	ND6	CDS	16594	16774	17112	-	3	G	A	transition	G	G	synonymous	NADH dehydrogenase subunit 6
KT361659	Amazona aestiva	ND6	CDS	16594	16780	17112	-	3	G	A	transition	G	G	synonymous	NADH dehydrogenase subunit 6
KT361659	Amazona aestiva	ND6	CDS	16594	16787	17112	-	2	G	A	transition	G	D	missense	NADH dehydrogenase subunit 6
KT361659	Amazona aestiva	ND6	CDS	16594	16798	17112	-	3	G	A	transition	W	W	synonymous	NADH dehydrogenase subunit 6
KT361659	Amazona aestiva	ND6	CDS	16594	16801	17112	-	3	T	C	transition	V	V	synonymous	NADH dehydrogenase subunit 6
KT361659	Amazona aestiva	ND6	CDS	16594	16806	17112	-	1	G	A	transition	V	I	missense	NADH dehydrogenase subunit 6
KT361659	Amazona aestiva	ND6	CDS	16594	16816	17112	-	3	T	C	transition	G	G	synonymous	NADH dehydrogenase subunit 6
KT361659	Amazona aestiva	ND6	CDS	16594	16819	17112	-	3	G	A	transition	V	V	synonymous	NADH dehydrogenase subunit 6
KT361659	Amazona aestiva	ND6	CDS	16594	16829	17112	-	2	T	C	transition	V	A	missense	NADH dehydrogenase subunit 6
KT361659	Amazona aestiva	ND6	CDS	16594	16839	17112	-	1	T	C	transition	L	L	synonymous	NADH dehydrogenase subunit 6
KT361659	Amazona aestiva	ND6	CDS	16594	16851	17112	-	1	G	A	transition	V	M	missense	NADH dehydrogenase subunit 6
KT361659	Amazona aestiva	ND6	CDS	16594	16857	17112	-	1	T	C	transition	Y	H	missense	NADH dehydrogenase subunit 6
KT361659	Amazona aestiva	ND6	CDS	16594	16888	17112	-	3	C	T	transition	A	A	synonymous	NADH dehydrogenase subunit 6
KT361659	Amazona aestiva	ND6	CDS	16594	16903	17112	-	3	T	C	transition	S	S	synonymous	NADH dehydrogenase subunit 6
KT361659	Amazona aestiva	ND6	CDS	16594	16962	17112	-	1	G	A	transition	V	M	missense	NADH dehydrogenase subunit 6
KT361659	Amazona aestiva	ND6	CDS	16594	17053	17112	-	3	G	A	transition	A	A	synonymous	NADH dehydrogenase subunit 6
KT361659	Amazona aestiva	ND6	CDS	16594	17056	17112	-	3	G	A	transition	V	V	synonymous	NADH dehydrogenase subunit 6
KT361659	Amazona aestiva	ND6	CDS	16594	17071	17112	-	3	A	G	transition	L	L	synonymous	NADH dehydrogenase subunit 6
KT361659	Amazona aestiva	ControlRegion2	Control Region	17183	17185	18853	+		C	T	transition	A	A		Control Region 2
KT361659	Amazona aestiva	ControlRegion2	Control Region	17183	17192	18853	+		G	A	transition	E	K		Control Region 2
KT361659	Amazona aestiva	ControlRegion2	Control Region	17183	17219	18853	+		T	C	transition	C	R		Control Region 2
KT361659	Amazona aestiva	ControlRegion2	Control Region	17183	17234	18853	+		T	C	transition	Y	H		Control Region 2
KT361659	Amazona aestiva	ControlRegion2	Control Region	17183	17250	18853	+		A	G	transition	E	G		Control Region 2
KT361659	Amazona aestiva	ControlRegion2	Control Region	17183	17263	18853	+		T	G	transversion	Y	*		Control Region 2
KT361659	Amazona aestiva	ControlRegion2	Control Region	17183	17273	18853	+		C	T	transition	L	L		Control Region 2
KT361659	Amazona aestiva	ControlRegion2	Control Region	17183	17289	18853	+		G	A	transition	*	K		Control Region 2
KT361659	Amazona aestiva	ControlRegion2	Control Region	17183	17293	18853	+		C	T	transition	D	D		Control Region 2
KT361659	Amazona aestiva	ControlRegion2	Control Region	17183	17309	18853	+		T	C	transition	C	H		Control Region 2
KT361659	Amazona aestiva	ControlRegion2	Control Region	17183	17310	18853	+		G	A	transition	C	H		Control Region 2
KT361659	Amazona aestiva	ControlRegion2	Control Region	17183	17326	18853	+		C	A	transversion	Y	*		Control Region 2
KT361659	Amazona aestiva	ControlRegion2	Control Region	17183	17328	18853	+		C	T	transition	S	L		Control Region 2
KT361659	Amazona aestiva	ControlRegion2	Control Region	17183	17353	18853	+		C	T	transition	V	V		Control Region 2
KT361659	Amazona aestiva	ControlRegion2	Control Region	17183	17370	18853	+		G	A	transition	*	K		Control Region 2
KT361659	Amazona aestiva	ControlRegion2	Control Region	17183	17380	18853	+		G	A	transition	W	W		Control Region 2
KT361659	Amazona aestiva	ControlRegion2	Control Region	17183	17383	18853	+		C	T	transition	I	I		Control Region 2
KT361659	Amazona aestiva	ControlRegion2	Control Region	17183	17385	18853	+		T	C	transition	M	T		Control Region 2
KT361659	Amazona aestiva	ControlRegion2	Control Region	17183	17392	18853	+		C	T	transition	S	S		Control Region 2
KT361659	Amazona aestiva	ControlRegion2	Control Region	17183	17395	18853	+		A	G	transition	*	*		Control Region 2

Genbank ID	Organism	Gene	Type	Start	Actual Position	End	Strand	Codon Position	A. aestiva	A. barbadensis	Type of Mutation	A. aestiva AA	A. barbadensis AA	Type of Mutation	Product
KT361659	Amazona aestiva	ControlRegion2	Control Region	17183	17396	18853	+		T	C	transition	L	L		Control Region 2
KT361659	Amazona aestiva	ControlRegion2	Control Region	17183	17409	18853	+		A	G	transition	K	*		Control Region 2
KT361659	Amazona aestiva	ControlRegion2	Control Region	17183	17412	18853	+		C	T	transition	T	M		Control Region 2
KT361659	Amazona aestiva	ControlRegion2	Control Region	17183	17441	18853	+		T	C	transition	F	L		Control Region 2
KT361659	Amazona aestiva	ControlRegion2	Control Region	17183	17443	18853	+		C	T	transition	F	L		Control Region 2
KT361659	Amazona aestiva	ControlRegion2	Control Region	17183	17461	18853	+		T	C	transition	L	L		Control Region 2
KT361659	Amazona aestiva	ControlRegion2	Control Region	17183	17462	18853	+		T	C	transition	L	L		Control Region 2
KT361659	Amazona aestiva	ControlRegion2	Control Region	17183	17465	18853	+		C	T	transition	H	Y		Control Region 2
KT361659	Amazona aestiva	ControlRegion2	Control Region	17183	17472	18853	+		G	A	transition	*	K		Control Region 2
KT361659	Amazona aestiva	ControlRegion2	Control Region	17183	17473	18853	+		A	G	transition	*	K		Control Region 2
KT361659	Amazona aestiva	ControlRegion2	Control Region	17183	17475	18853	+		G	C	transversion	W	S		Control Region 2
KT361659	Amazona aestiva	ControlRegion2	Control Region	17183	17480	18853	+		C	T	transition	L	F		Control Region 2
KT361659	Amazona aestiva	ControlRegion2	Control Region	17183	17484	18853	+		C	T	transition	S	F		Control Region 2
KT361659	Amazona aestiva	ControlRegion2	Control Region	17183	17485	18853	+		T	C	transition	S	F		Control Region 2
KT361659	Amazona aestiva	ControlRegion2	Control Region	17183	17487	18853	+		T	A	transversion	V	D		Control Region 2
KT361659	Amazona aestiva	ControlRegion2	Control Region	17183	17492	18853	+		T	A	transversion	S	T		Control Region 2
KT361659	Amazona aestiva	ControlRegion2	Control Region	17183	17503	18853	+		T	C	transition	L	L		Control Region 2
KT361659	Amazona aestiva	ControlRegion2	Control Region	17183	17520	18853	+		T	C	transition	L	P		Control Region 2
KT361659	Amazona aestiva	ControlRegion2	Control Region	17183	17913	18853	+		A	G	transition	Y	C		Control Region 2
KT361659	Amazona aestiva	ControlRegion2	Control Region	17183	18061	18853	+		C	G	transversion	R	R		Control Region 2
KT361659	Amazona aestiva	ControlRegion2	Control Region	17183	18065	18853	+		C	.	INDEL	R			Control Region 2
KT361659	Amazona aestiva	ControlRegion2	Control Region	17183	18143	18853	+		A	G	transition	I	V		Control Region 2
KT361659	Amazona aestiva	ControlRegion2	Control Region	17183	18172	18853	+		C	T	transition	F	F		Control Region 2
KT361659	Amazona aestiva	ControlRegion2	Control Region	17183	18173	18853	+		A	G	transition	S	G		Control Region 2
KT361659	Amazona aestiva	ControlRegion2	Control Region	17183	18181	18853	+		C	T	transition	T	T		Control Region 2
KT361659	Amazona aestiva	ControlRegion2	Control Region	17183	18182	18853	+		G	T	transversion	V	L		Control Region 2
KT361659	Amazona aestiva	ControlRegion2	Control Region	17183	18184	18853	+		A	G	transition	V	L		Control Region 2
KT361659	Amazona aestiva	ControlRegion2	Control Region	17183	18208	18853	+		C	T	transition	H	H		Control Region 2
KT361659	Amazona aestiva	ControlRegion2	Control Region	17183	18292	18853	+		C	T	transition	C	C		Control Region 2
KT361659	Amazona aestiva	ControlRegion2	Control Region	17183	18457	18853	+		.	T	INDEL		P		Control Region 2
KT361659	Amazona aestiva	ControlRegion2	Control Region	17183	18538	18853	+		.	C	INDEL		F		Control Region 2
KT361659	Amazona aestiva	ControlRegion2	Control Region	17183	18538	18853	+		.	A	INDEL		S		Control Region 2
KT361659	Amazona aestiva	ControlRegion2	Control Region	17183	18538	18853	+		.	T	INDEL		H		Control Region 2
KT361659	Amazona aestiva	ControlRegion2	Control Region	17183	18538	18853	+		.	T	INDEL		I		Control Region 2
KT361659	Amazona aestiva	ControlRegion2	Control Region	17183	18540	18853	+		.	T	INDEL		V		Control Region 2
KT361659	Amazona aestiva	ControlRegion2	Control Region	17183	18540	18853	+		.	T	INDEL		F		Control Region 2
KT361659	Amazona aestiva	ControlRegion2	Control Region	17183	18540	18853	+		.	C	INDEL		S		Control Region 2
KT361659	Amazona aestiva	ControlRegion2	Control Region	17183	18540	18853	+		.	A	INDEL		H		Control Region 2
KT361659	Amazona aestiva	ControlRegion2	Control Region	17183	18556	18853	+		G	A	transition	S	S		Control Region 2
KT361659	Amazona aestiva	ControlRegion2	Control Region	17183	18772	18853	+		A	.	INDEL	S			Control Region 2
KT361659	Amazona aestiva	ControlRegion2	Control Region	17183	18777	18853	+		A	.	INDEL	D			Control Region 2

Genbank ID	Organism	Gene	Type	Start	Actual Position	End	Strand	Codon Position	A. aestiva	A. barbadensis	Type of Mutation	A. aestiva AA	A. barbadensis AA	Type of Mutation	Product
KT361659	Amazona aestiva	ControlRegion2	Control Region	17183	18778	18853	+		C	.	INDEL	D			Control Region 2
KT361659	Amazona aestiva	ControlRegion2	Control Region	17183	18780	18853	+		A	G	transition	H	R		Control Region 2
KT361659	Amazona aestiva	ControlRegion2	Control Region	17183	18786	18853	+		.	T	INDEL				Control Region 2
