# Supplementary material for: Comparative mitogenomic analyses of Amazona parrots and Psittaciformes
Source: Genet Mol Biol. 2018 Jul-Sep;41(3):593–604. doi: 10.1590/1678-4685-GMB-2017-0023 (PMC6136379; doi:10.1590/1678-4685-GMB-2017-0023)
Supplement: Table S6 - [file 1415-4757-GMB-41-03-2017-0023-20180716-suppl6.pdf]

Supplementary Material to "Comparative mitogenomic analyses of *Amazona* parrots and Psittaciformes"

Table S6 - Polymorphisms found in the whole mitogenome alignment of *Amazona aestiva* and *Amazona ochrocephala*.

Genbank ID	Organism	Gene	Type	Start	Actual Position	End	Strand	Codon Position	A. aestiva	A. barbadensis	Type of Mutation	A. aestiva AA	A. barbadensis AA	Type of Mutation	Product
KT361659	Amazona aestiva	s-rRNA	rRNA	66	284	1033	+		A	G	transition				12S ribosomal RNA
KT361659	Amazona aestiva	s-rRNA	rRNA	66	428	1033	+		A	C	transversion				12S ribosomal RNA
KT361659	Amazona aestiva	l-rRNA	rRNA	1106	1146	2673	+		C	T	transition				16S ribosomal RNA
KT361659	Amazona aestiva	l-rRNA	rRNA	1106	1446	2673	+		C	.	INDEL				16S ribosomal RNA
KT361659	Amazona aestiva	l-rRNA	rRNA	1106	1485	2673	+		A	G	transition				16S ribosomal RNA
KT361659	Amazona aestiva	l-rRNA	rRNA	1106	1792	2673	+		A	G	transition				16S ribosomal RNA
KT361659	Amazona aestiva	l-rRNA	rRNA	1106	1914	2673	+		T	C	transition				16S ribosomal RNA
KT361659	Amazona aestiva	l-rRNA	rRNA	1106	2583	2673	+		T	C	transition				16S ribosomal RNA
KT361659	Amazona aestiva	l-rRNA	rRNA	1106	2647	2673	+		T	C	transition				16S ribosomal RNA
KT361659	Amazona aestiva	ND1	CDS	2754	2816	3733	+	3	C	T	transition	I	I	synonymous	NADH dehydrogenase subunit 1
KT361659	Amazona aestiva	ND1	CDS	2754	2864	3733	+	3	C	T	transition	S	S	synonymous	NADH dehydrogenase subunit 1
KT361659	Amazona aestiva	ND1	CDS	2754	2981	3733	+	3	G	A	transition	P	P	synonymous	NADH dehydrogenase subunit 1
KT361659	Amazona aestiva	ND1	CDS	2754	3110	3733	+	3	G	A	transition	L	L	synonymous	NADH dehydrogenase subunit 1
KT361659	Amazona aestiva	ND2	CDS	3951	4019	4991	+	3	C	T	transition	S	S	synonymous	NADH dehydrogenase subunit 2
KT361659	Amazona aestiva	ND2	CDS	3951	4209	4991	+	1	A	G	transition	T	A	missense	NADH dehydrogenase subunit 2
KT361659	Amazona aestiva	ND2	CDS	3951	4438	4991	+	2	C	T	transition	T	I	missense	NADH dehydrogenase subunit 2
KT361659	Amazona aestiva	ND2	CDS	3951	4508	4991	+	3	T	C	transition	H	H	synonymous	NADH dehydrogenase subunit 2
KT361659	Amazona aestiva	ND2	CDS	3951	4576	4991	+	2	T	C	transition	I	T	missense	NADH dehydrogenase subunit 2
KT361659	Amazona aestiva	ND2	CDS	3951	4805	4991	+	3	A	G	transition	A	A	synonymous	NADH dehydrogenase subunit 2
KT361659	Amazona aestiva	ND2	CDS	3951	4967	4991	+	3	T	C	transition	S	S	synonymous	NADH dehydrogenase

Genbank ID	Organism	Gene	Type	Start	Actual Position	End	Strand	Codon Position	A. aestiva	A. barbadensis	Type of Mutation	A. aestiva AA	A. barbadensis AA	Type of Mutation	Product
															subunit 2
KT361659	Amazona aestiva	COX1	CDS	5354	5743	6901	+	3	C	T	transition	P	P	synonymous	cytochrome c oxidase subunit I
KT361659	Amazona aestiva	COX1	CDS	5354	5950	6901	+	3	G	A	transition	L	L	synonymous	cytochrome c oxidase subunit I
KT361659	Amazona aestiva	COX1	CDS	5354	6082	6901	+	3	G	A	transition	V	V	synonymous	cytochrome c oxidase subunit I
KT361659	Amazona aestiva	COX1	CDS	5354	6343	6901	+	3	G	A	transition	G	G	synonymous	cytochrome c oxidase subunit I
KT361659	Amazona aestiva	COX1	CDS	5354	6493	6901	+	3	T	C	transition	V	V	synonymous	cytochrome c oxidase subunit I
KT361659	Amazona aestiva	COX1	CDS	5354	6574	6901	+	3	G	A	transition	Q	Q	synonymous	cytochrome c oxidase subunit I
KT361659	Amazona aestiva	COX1	CDS	5354	6661	6901	+	3	A	G	transition	M	M	synonymous	cytochrome c oxidase subunit I
KT361659	Amazona aestiva	COX1	CDS	5354	6754	6901	+	3	A	G	transition	M	M	synonymous	cytochrome c oxidase subunit I
KT361659	Amazona aestiva	tRNA-Asp	tRNA	6971	7023	7039	+		G	A	transition				transfer RNA aspartate
KT361659	Amazona aestiva	COX2	CDS	7042	7119	7725	+	3	T	C	transition	H	H	synonymous	cytochrome c oxidase subunit II
KT361659	Amazona aestiva	COX2	CDS	7042	7134	7725	+	3	T	C	transition	A	A	synonymous	cytochrome c oxidase subunit II
KT361659	Amazona aestiva	COX2	CDS	7042	7380	7725	+	3	G	A	transition	K	K	synonymous	cytochrome c oxidase subunit II
KT361659	Amazona aestiva	COX2	CDS	7042	7498	7725	+	1	A	G	transition	I	V	missense	cytochrome c oxidase subunit II
KT361659	Amazona aestiva	ATP8	CDS	7797	7940	7964	+	3	A	G	transition	K	K	synonymous	ATP synthase F0 subunit 8
KT361659	Amazona aestiva	ATP8	CDS	7797	7948	7964	+	2	T	C	transition	I	T	missense	ATP synthase F0 subunit 8
KT361659	Amazona aestiva	ATP6	CDS	7955	8009	8638	+	1	T	C	transition	L	L	synonymous	ATP synthase F0 subunit 6
KT361659	Amazona aestiva	ATP6	CDS	7955	8059	8638	+	3	T	C	transition	N	N	synonymous	ATP synthase F0 subunit 6
KT361659	Amazona aestiva	ATP6	CDS	7955	8180	8638	+	1	T	C	transition	L	L	synonymous	ATP synthase F0 subunit 6
KT361659	Amazona aestiva	ATP6	CDS	7955	8203	8638	+	3	T	C	transition	I	I	synonymous	ATP synthase F0 subunit 6
KT361659	Amazona aestiva	ATP6	CDS	7955	8236	8638	+	3	T	C	transition	T	T	synonymous	ATP synthase F0 subunit 6
KT361659	Amazona aestiva	ATP6	CDS	7955	8425	8638	+	3	G	A	transition	L	L	synonymous	ATP synthase F0 subunit 6
KT361659	Amazona aestiva	COX3	CDS	8638	8685	9421	+	3	A	G	transition	W	W	synonymous	cytochrome c oxidase subunit III
KT361659	Amazona aestiva	COX3	CDS	8638	8715	9421	+	3	A	G	transition	L	L	synonymous	cytochrome c oxidase subunit III
KT361659	Amazona aestiva	COX3	CDS	8638	9104	9421	+	2	A	G	transition	Q	R	missense	cytochrome c oxidase subunit III
KT361659	Amazona aestiva	COX3	CDS	8638	9207	9421	+	3	C	T	transition	D	D	synonymous	cytochrome c oxidase subunit III
KT361659	Amazona aestiva	COX3	CDS	8638	9231	9421	+	3	C	T	transition	F	F	synonymous	cytochrome c oxidase subunit III
KT361659	Amazona aestiva	COX3	CDS	8638	9255	9421	+	3	C	T	transition	L	L	synonymous	cytochrome c oxidase subunit III
KT361659	Amazona aestiva	COX3	CDS	8638	9387	9421	+	3	A	G	transition	L	L	synonymous	cytochrome c oxidase subunit III

Genbank ID	Organism	Gene	Type	Start	Actual Position	End	Strand	Codon Position	A. aestiva	A. barbadensis	Type of Mutation	A. aestiva AA	A. barbadensis AA	Type of Mutation	Product
KT361659	Amazona aestiva	COX3	CDS	8638	9390	9421	+	3	T	C	transition	F	F	synonymous	cytochrome c oxidase subunit III
KT361659	Amazona aestiva	ND3	CDS	9490	9691	9841	+	3	C	T	transition	D	D	synonymous	NADH dehydrogenase subunit 3
KT361659	Amazona aestiva	ND3	CDS	9490	9718	9841	+	3	A	G	transition	L	L	synonymous	NADH dehydrogenase subunit 3
KT361659	Amazona aestiva	ND4L	CDS	9911	9994	10207	+	3	C	T	transition	S	S	synonymous	NADH dehydrogenase subunit 4L
KT361659	Amazona aestiva	ND4L	CDS	9911	10091	10207	+	1	A	G	transition	I	V	missense	NADH dehydrogenase subunit 4L
KT361659	Amazona aestiva	ND4	CDS	10201	10248	11593	+	3	C	T	transition	L	L	synonymous	NADH dehydrogenase subunit 4
KT361659	Amazona aestiva	ND4	CDS	10201	10311	11593	+	3	G	A	transition	L	L	synonymous	NADH dehydrogenase subunit 4
KT361659	Amazona aestiva	ND4	CDS	10201	10640	11593	+	2	T	C	transition	I	T	missense	NADH dehydrogenase subunit 4
KT361659	Amazona aestiva	ND4	CDS	10201	10774	11593	+	1	A	G	transition	K	E	missense	NADH dehydrogenase subunit 4
KT361659	Amazona aestiva	ND4	CDS	10201	10963	11593	+	1	A	G	transition	N	D	missense	NADH dehydrogenase subunit 4
KT361659	Amazona aestiva	ND4	CDS	10201	11011	11593	+	1	T	C	transition	L	L	synonymous	NADH dehydrogenase subunit 4
KT361659	Amazona aestiva	ND4	CDS	10201	11139	11593	+	3	T	C	transition	S	S	synonymous	NADH dehydrogenase subunit 4
KT361659	Amazona aestiva	ND4	CDS	10201	11172	11593	+	3	T	C	transition	H	H	synonymous	NADH dehydrogenase subunit 4
KT361659	Amazona aestiva	ND4	CDS	10201	11280	11593	+	3	G	A	transition	M	M	synonymous	NADH dehydrogenase subunit 4
KT361659	Amazona aestiva	ND5	CDS	11799	11858	13622	+	3	A	G	transition	T	T	synonymous	NADH dehydrogenase subunit 5
KT361659	Amazona aestiva	ND5	CDS	11799	11972	13622	+	3	C	T	transition	S	S	synonymous	NADH dehydrogenase subunit 5
KT361659	Amazona aestiva	ND5	CDS	11799	12140	13622	+	3	G	A	transition	P	P	synonymous	NADH dehydrogenase subunit 5
KT361659	Amazona aestiva	ND5	CDS	11799	12182	13622	+	3	T	C	transition	I	I	synonymous	NADH dehydrogenase subunit 5
KT361659	Amazona aestiva	ND5	CDS	11799	12506	13622	+	3	T	C	transition	A	A	synonymous	NADH dehydrogenase subunit 5
KT361659	Amazona aestiva	ND5	CDS	11799	12551	13622	+	3	C	T	transition	S	S	synonymous	NADH dehydrogenase subunit 5

Genbank ID	Organism	Gene	Type	Start	Actual Position	End	Strand	Codon Position	A. aestiva	A. barbadensis	Type of Mutation	A. aestiva AA	A. barbadensis AA	Type of Mutation	Product
KT361659	Amazona aestiva	ND5	CDS	11799	12735	13622	+	1	T	C	transition	L	L	synonymous	NADH dehydrogenase subunit 5
KT361659	Amazona aestiva	ND5	CDS	11799	12833	13622	+	3	G	A	transition	G	G	synonymous	NADH dehydrogenase subunit 5
KT361659	Amazona aestiva	ND5	CDS	11799	13007	13622	+	3	C	T	transition	S	S	synonymous	NADH dehydrogenase subunit 5
KT361659	Amazona aestiva	ND5	CDS	11799	13122	13622	+	1	G	A	transition	V	I	missense	NADH dehydrogenase subunit 5
KT361659	Amazona aestiva	ND5	CDS	11799	13343	13622	+	3	C	T	transition	P	P	synonymous	NADH dehydrogenase subunit 5
KT361659	Amazona aestiva	ND5	CDS	11799	13385	13622	+	3	T	C	transition	Y	Y	synonymous	NADH dehydrogenase subunit 5
KT361659	Amazona aestiva	ND5	CDS	11799	13444	13622	+	2	C	T	transition	T	I	missense	NADH dehydrogenase subunit 5
KT361659	Amazona aestiva	ND5	CDS	11799	13460	13622	+	3	A	G	transition	M	M	synonymous	NADH dehydrogenase subunit 5
KT361659	Amazona aestiva	ND5	CDS	11799	13505	13622	+	3	T	C	transition	N	N	synonymous	NADH dehydrogenase subunit 5
KT361659	Amazona aestiva	ND5	CDS	11799	13547	13622	+	3	G	A	transition	K	K	synonymous	NADH dehydrogenase subunit 5
KT361659	Amazona aestiva	ND5	CDS	11799	13601	13622	+	3	A	G	transition	L	L	synonymous	NADH dehydrogenase subunit 5
KT361659	Amazona aestiva	CYTB	CDS	13622	13777	14761	+	3	A	G	transition	L	L	synonymous	cytochrome b
KT361659	Amazona aestiva	CYTB	CDS	13622	13936	14761	+	3	T	C	transition	Y	Y	synonymous	cytochrome b
KT361659	Amazona aestiva	CYTB	CDS	13622	14053	14761	+	3	C	T	transition	A	A	synonymous	cytochrome b
KT361659	Amazona aestiva	CYTB	CDS	13622	14055	14761	+	2	C	N	Undetermined	T	Possibly an T	Possibly synonymous	cytochrome b
KT361659	Amazona aestiva	CYTB	CDS	13622	14080	14761	+	3	T	C	transition	A	A	synonymous	cytochrome b
KT361659	Amazona aestiva	CYTB	CDS	13622	14155	14761	+	3	A	G	transition	R	R	synonymous	cytochrome b
KT361659	Amazona aestiva	CYTB	CDS	13622	14254	14761	+	3	C	T	transition	G	G	synonymous	cytochrome b
KT361659	Amazona aestiva	CYTB	CDS	13622	14398	14761	+	3	A	G	transition	P	P	synonymous	cytochrome b
KT361659	Amazona aestiva	CYTB	CDS	13622	14458	14761	+	3	C	T	transition	Y	Y	synonymous	cytochrome b
KT361659	Amazona aestiva	CYTB	CDS	13622	14491	14761	+	3	G	A	transition	G	G	synonymous	cytochrome b
KT361659	Amazona aestiva	pseudoND6	pseudogene	14831	14864	14890	+		A	G	transition				pseudoND6
KT361659	Amazona aestiva	pseudoND6	pseudogene	14831	14879	14890	+		T	C	transition				pseudoND6
KT361659	Amazona aestiva	ControlRegion1	Control Region	14957	14968	16521	+		G	A	transition				Control Region 1
KT361659	Amazona aestiva	ControlRegion1	Control Region	14957	14980	16521	+		.	C	INDEL				Control Region 1
KT361659	Amazona aestiva	ControlRegion1	Control Region	14957	14992	16521	+		T	C	transition				Control Region 1
KT361659	Amazona aestiva	ControlRegion1	Control Region	14957	15066	16521	+		C	T	transition				Control Region 1
KT361659	Amazona aestiva	ControlRegion1	Control Region	14957	15074	16521	+		T	C	transition				Control Region 1

Genbank ID	Organism	Gene	Type	Start	Actual Position	End	Strand	Codon Position	A. aestiva	A. barbadensis	Type of Mutation	A. aestiva AA	A. barbadensis AA	Type of Mutation	Product
KT361659	Amazona aestiva	ControlRegion1	Control Region	14957	15082	16521	+		T	C	transition				Control Region 1
KT361659	Amazona aestiva	ControlRegion1	Control Region	14957	15126	16521	+		C	T	transition				Control Region 1
KT361659	Amazona aestiva	ControlRegion1	Control Region	14957	15138	16521	+		G	A	transition				Control Region 1
KT361659	Amazona aestiva	ControlRegion1	Control Region	14957	15143	16521	+		G	A	transition				Control Region 1
KT361659	Amazona aestiva	ControlRegion1	Control Region	14957	15156	16521	+		C	T	transition				Control Region 1
KT361659	Amazona aestiva	ControlRegion1	Control Region	14957	15165	16521	+		C	T	transition				Control Region 1
KT361659	Amazona aestiva	ControlRegion1	Control Region	14957	15169	16521	+		T	C	transition				Control Region 1
KT361659	Amazona aestiva	ControlRegion1	Control Region	14957	15214	16521	+		T	C	transition				Control Region 1
KT361659	Amazona aestiva	ControlRegion1	Control Region	14957	15217	16521	+		T	C	transition				Control Region 1
KT361659	Amazona aestiva	ControlRegion1	Control Region	14957	15219	16521	+		G	A	transition				Control Region 1
KT361659	Amazona aestiva	ControlRegion1	Control Region	14957	15234	16521	+		T	C	transition				Control Region 1
KT361659	Amazona aestiva	ControlRegion1	Control Region	14957	15235	16521	+		T	C	transition				Control Region 1
KT361659	Amazona aestiva	ControlRegion1	Control Region	14957	15238	16521	+		C	T	transition				Control Region 1
KT361659	Amazona aestiva	ControlRegion1	Control Region	14957	15244	16521	+		A	G	transition				Control Region 1
KT361659	Amazona aestiva	ControlRegion1	Control Region	14957	15245	16521	+		G	A	transition				Control Region 1
KT361659	Amazona aestiva	ControlRegion1	Control Region	14957	15258	16521	+		T	C	transition				Control Region 1
KT361659	Amazona aestiva	ControlRegion1	Control Region	14957	15287	16521	+		T	C	transition				Control Region 1
KT361659	Amazona aestiva	ControlRegion1	Control Region	14957	15293	16521	+		T	C	transition				Control Region 1
KT361659	Amazona aestiva	ControlRegion1	Control Region	14957	15834	16521	+		C	G	transversion				Control Region 1
KT361659	Amazona aestiva	ControlRegion1	Control Region	14957	15894	16521	+		G	A	transition				Control Region 1
KT361659	Amazona aestiva	ControlRegion1	Control Region	14957	15916	16521	+		A	G	transition				Control Region 1
KT361659	Amazona aestiva	ControlRegion1	Control Region	14957	16228	16521	+		.	C	INDEL				Control Region 1
KT361659	Amazona aestiva	ControlRegion1	Control Region	14957	16272	16521	+		.	T	INDEL				Control Region 1
KT361659	Amazona aestiva	ControlRegion1	Control Region	14957	16272	16521	+		.	G	INDEL				Control Region 1
KT361659	Amazona aestiva	ControlRegion1	Control Region	14957	16272	16521	+		.	T	INDEL				Control Region 1
KT361659	Amazona aestiva	ControlRegion1	Control Region	14957	16272	16521	+		.	T	INDEL				Control Region 1
KT361659	Amazona aestiva	ControlRegion1	Control Region	14957	16272	16521	+		.	G	INDEL				Control Region 1
KT361659	Amazona aestiva	ControlRegion1	Control Region	14957	16272	16521	+		.	T	INDEL				Control Region 1
KT361659	Amazona aestiva	ControlRegion1	Control Region	14957	16272	16521	+		.	T	INDEL				Control Region 1
KT361659	Amazona aestiva	ControlRegion1	Control Region	14957	16272	16521	+		.	T	INDEL				Control Region 1
KT361659	Amazona aestiva	ControlRegion1	Control Region	14957	16272	16521	+		.	C	INDEL				Control Region 1
KT361659	Amazona aestiva	ControlRegion1	Control Region	14957	16272	16521	+		.	G	INDEL				Control Region 1
KT361659	Amazona aestiva	ControlRegion1	Control Region	14957	16272	16521	+		.	T	INDEL				Control Region 1
KT361659	Amazona aestiva	ControlRegion1	Control Region	14957	16272	16521	+		.	T	INDEL				Control Region 1
KT361659	Amazona aestiva	ControlRegion1	Control Region	14957	16272	16521	+		.	G	INDEL				Control Region 1
KT361659	Amazona aestiva	ControlRegion1	Control Region	14957	16272	16521	+		.	T	INDEL				Control Region 1
KT361659	Amazona aestiva	ControlRegion1	Control Region	14957	16272	16521	+		.	T	INDEL				Control Region 1
KT361659	Amazona aestiva	ControlRegion1	Control Region	14957	16272	16521	+		.	T	INDEL				Control Region 1
KT361659	Amazona aestiva	ControlRegion1	Control Region	14957	16272	16521	+		.	C	INDEL				Control Region 1
KT361659	Amazona aestiva	ControlRegion1	Control Region	14957	16272	16521	+		.	G	INDEL				Control Region 1
KT361659	Amazona aestiva	ControlRegion1	Control Region	14957	16272	16521	+		.	T	INDEL				Control Region 1

Genbank ID	Organism	Gene	Type	Start	Actual Position	End	Strand	Codon Position	A. aestiva	A. barbadensis	Type of Mutation	A. aestiva AA	A. barbadensis AA	Type of Mutation	Product
KT361659	Amazona aestiva	ControlRegion1	Control Region	14957	16272	16521	+		.	T	INDEL				Control Region 1
KT361659	Amazona aestiva	ControlRegion1	Control Region	14957	16272	16521	+		.	G	INDEL				Control Region 1
KT361659	Amazona aestiva	ControlRegion1	Control Region	14957	16272	16521	+		.	T	INDEL				Control Region 1
KT361659	Amazona aestiva	ControlRegion1	Control Region	14957	16272	16521	+		.	T	INDEL				Control Region 1
KT361659	Amazona aestiva	ControlRegion1	Control Region	14957	16272	16521	+		.	T	INDEL				Control Region 1
KT361659	Amazona aestiva	ControlRegion1	Control Region	14957	16272	16521	+		.	C	INDEL				Control Region 1
KT361659	Amazona aestiva	ControlRegion1	Control Region	14957	16272	16521	+		.	G	INDEL				Control Region 1
KT361659	Amazona aestiva	ControlRegion1	Control Region	14957	16277	16521	+		.	T	INDEL				Control Region 1
KT361659	Amazona aestiva	ControlRegion1	Control Region	14957	16277	16521	+		.	C	INDEL				Control Region 1
KT361659	Amazona aestiva	ControlRegion1	Control Region	14957	16358	16521	+		G	A	transition				Control Region 1
KT361659	Amazona aestiva	ControlRegion1	Control Region	14957	16370	16521	+		T	C	transition				Control Region 1
KT361659	Amazona aestiva	ControlRegion1	Control Region	14957	16373	16521	+		C	T	transition				Control Region 1
KT361659	Amazona aestiva	ControlRegion1	Control Region	14957	16387	16521	+		A	C	transversion				Control Region 1
KT361659	Amazona aestiva	ControlRegion1	Control Region	14957	16446	16521	+		G	A	transition				Control Region 1
KT361659	Amazona aestiva	ControlRegion1	Control Region	14957	16449	16521	+		A	G	transition				Control Region 1
KT361659	Amazona aestiva	ControlRegion1	Control Region	14957	16475	16521	+		T	C	transition				Control Region 1
KT361659	Amazona aestiva	tRNA-Pro	tRNA	16522	16581	16590	-		.	A	INDEL				transfer RNA proline
KT361659	Amazona aestiva	tRNA-Pro	tRNA	16522	16588	16590	-		G	A	transition				transfer RNA proline
KT361659	Amazona aestiva	ND6	CDS	16594	16603	17112	-	3	G	A	transition	R	R	synonymous	NADH dehydrogenase subunit 6
KT361659	Amazona aestiva	ND6	CDS	16594	16614	17112	-	1	G	T	transversion	G	W	missense	NADH dehydrogenase subunit 6
KT361659	Amazona aestiva	ND6	CDS	16594	16659	17112	-	1	T	G	transversion	L	V	missense	NADH dehydrogenase subunit 6
KT361659	Amazona aestiva	ND6	CDS	16594	16728	17112	-	1	T	N	Undetermined	F	Possibly an F	Possibly synonymous	NADH dehydrogenase subunit 6
KT361659	Amazona aestiva	ND6	CDS	16594	16750	17112	-	3	A	T	transversion	G	G	synonymous	NADH dehydrogenase subunit 6
KT361659	Amazona aestiva	ND6	CDS	16594	16757	17112	-	2	A	G	transition	N	S	missense	NADH dehydrogenase subunit 6
KT361659	Amazona aestiva	ND6	CDS	16594	16780	17112	-	3	G	A	transition	G	G	synonymous	NADH dehydrogenase subunit 6
KT361659	Amazona aestiva	ND6	CDS	16594	16819	17112	-	3	G	A	transition	V	V	synonymous	NADH dehydrogenase subunit 6
KT361659	Amazona aestiva	ND6	CDS	16594	16851	17112	-	1	G	A	transition	V	M	missense	NADH dehydrogenase subunit 6
KT361659	Amazona aestiva	ND6	CDS	16594	16930	17112	-	3	T	C	transition	G	G	synonymous	NADH dehydrogenase subunit 6
KT361659	Amazona aestiva	ND6	CDS	16594	16984	17112	-	3	G	A	transition	L	L	synonymous	NADH dehydrogenase subunit 6

Genbank ID	Organism	Gene	Type	Start	Actual Position	End	Strand	Codon Position	A. aestiva	A. barbadensis	Type of Mutation	A. aestiva AA	A. barbadensis AA	Type of Mutation	Product
KT361659	Amazona aestiva	ND6	CDS	16594	17044	17112	-	3	T	C	transition	P	P	synonymous	NADH dehydrogenase subunit 6
KT361659	Amazona aestiva	ND6	CDS	16594	17071	17112	-	3	A	G	transition	L	L	synonymous	NADH dehydrogenase subunit 6
KT361659	Amazona aestiva	ControlRegion2	Control Region	17183	17183	18853	+		G	.	INDEL				Control Region 2
KT361659	Amazona aestiva	ControlRegion2	Control Region	17183	17219	18853	+		T	C	transition				Control Region 2
KT361659	Amazona aestiva	ControlRegion2	Control Region	17183	17302	18853	+		T	C	transition				Control Region 2
KT361659	Amazona aestiva	ControlRegion2	Control Region	17183	17310	18853	+		G	A	transition				Control Region 2
KT361659	Amazona aestiva	ControlRegion2	Control Region	17183	17311	18853	+		C	T	transition				Control Region 2
KT361659	Amazona aestiva	ControlRegion2	Control Region	17183	17338	18853	+		T	C	transition				Control Region 2
KT361659	Amazona aestiva	ControlRegion2	Control Region	17183	17348	18853	+		C	T	transition				Control Region 2
KT361659	Amazona aestiva	ControlRegion2	Control Region	17183	17365	18853	+		G	A	transition				Control Region 2
KT361659	Amazona aestiva	ControlRegion2	Control Region	17183	17370	18853	+		G	A	transition				Control Region 2
KT361659	Amazona aestiva	ControlRegion2	Control Region	17183	17383	18853	+		C	T	transition				Control Region 2
KT361659	Amazona aestiva	ControlRegion2	Control Region	17183	17392	18853	+		C	T	transition				Control Region 2
KT361659	Amazona aestiva	ControlRegion2	Control Region	17183	17396	18853	+		T	C	transition				Control Region 2
KT361659	Amazona aestiva	ControlRegion2	Control Region	17183	17441	18853	+		T	C	transition				Control Region 2
KT361659	Amazona aestiva	ControlRegion2	Control Region	17183	17444	18853	+		T	C	transition				Control Region 2
KT361659	Amazona aestiva	ControlRegion2	Control Region	17183	17446	18853	+		G	A	transition				Control Region 2
KT361659	Amazona aestiva	ControlRegion2	Control Region	17183	17461	18853	+		T	C	transition				Control Region 2
KT361659	Amazona aestiva	ControlRegion2	Control Region	17183	17462	18853	+		T	C	transition				Control Region 2
KT361659	Amazona aestiva	ControlRegion2	Control Region	17183	17465	18853	+		C	T	transition				Control Region 2
KT361659	Amazona aestiva	ControlRegion2	Control Region	17183	17471	18853	+		A	G	transition				Control Region 2
KT361659	Amazona aestiva	ControlRegion2	Control Region	17183	17472	18853	+		G	A	transition				Control Region 2
KT361659	Amazona aestiva	ControlRegion2	Control Region	17183	17480	18853	+		C	T	transition				Control Region 2
KT361659	Amazona aestiva	ControlRegion2	Control Region	17183	17485	18853	+		T	C	transition				Control Region 2
KT361659	Amazona aestiva	ControlRegion2	Control Region	17183	17514	18853	+		T	C	transition				Control Region 2
KT361659	Amazona aestiva	ControlRegion2	Control Region	17183	17520	18853	+		T	C	transition				Control Region 2
KT361659	Amazona aestiva	ControlRegion2	Control Region	17183	18061	18853	+		C	G	transversion				Control Region 2
KT361659	Amazona aestiva	ControlRegion2	Control Region	17183	18121	18853	+		G	A	transition				Control Region 2
KT361659	Amazona aestiva	ControlRegion2	Control Region	17183	18143	18853	+		A	G	transition				Control Region 2
KT361659	Amazona aestiva	ControlRegion2	Control Region	17183	18455	18853	+		.	C	INDEL				Control Region 2
KT361659	Amazona aestiva	ControlRegion2	Control Region	17183	18540	18853	+		G	A	transition				Control Region 2
KT361659	Amazona aestiva	ControlRegion2	Control Region	17183	18556	18853	+		G	A	transition				Control Region 2
KT361659	Amazona aestiva	ControlRegion2	Control Region	17183	18729	18853	+		.	T	INDEL				Control Region 2
KT361659	Amazona aestiva	ControlRegion2	Control Region	17183	18729	18853	+		.	C	INDEL				Control Region 2
KT361659	Amazona aestiva	ControlRegion2	Control Region	17183	18729	18853	+		.	A	INDEL				Control Region 2
KT361659	Amazona aestiva	ControlRegion2	Control Region	17183	18729	18853	+		.	T	INDEL				Control Region 2
KT361659	Amazona aestiva	ControlRegion2	Control Region	17183	18729	18853	+		.	T	INDEL				Control Region 2
KT361659	Amazona aestiva	ControlRegion2	Control Region	17183	18729	18853	+		.	G	INDEL				Control Region 2
KT361659	Amazona aestiva	ControlRegion2	Control Region	17183	18729	18853	+		.	T	INDEL				Control Region 2

Genbank ID	Organism	Gene	Type	Start	Actual Position	End	Strand	Codon Position	A. aestiva	A. barbadensis	Type of Mutation	A. aestiva AA	A. barbadensis AA	Type of Mutation	Product
KT361659	Amazona aestiva	ControlRegion2	Control Region	17183	18729	18853	+		.	T	INDEL				Control Region 2
KT361659	Amazona aestiva	ControlRegion2	Control Region	17183	18729	18853	+		.	C	INDEL				Control Region 2
KT361659	Amazona aestiva	ControlRegion2	Control Region	17183	18729	18853	+		.	A	INDEL				Control Region 2
KT361659	Amazona aestiva	ControlRegion2	Control Region	17183	18729	18853	+		.	T	INDEL				Control Region 2
KT361659	Amazona aestiva	ControlRegion2	Control Region	17183	18729	18853	+		.	T	INDEL				Control Region 2
KT361659	Amazona aestiva	ControlRegion2	Control Region	17183	18729	18853	+		.	G	INDEL				Control Region 2
KT361659	Amazona aestiva	ControlRegion2	Control Region	17183	18729	18853	+		.	T	INDEL				Control Region 2
KT361659	Amazona aestiva	ControlRegion2	Control Region	17183	18729	18853	+		.	T	INDEL				Control Region 2
KT361659	Amazona aestiva	ControlRegion2	Control Region	17183	18729	18853	+		.	C	INDEL				Control Region 2
KT361659	Amazona aestiva	ControlRegion2	Control Region	17183	18729	18853	+		.	A	INDEL				Control Region 2
KT361659	Amazona aestiva	ControlRegion2	Control Region	17183	18729	18853	+		.	T	INDEL				Control Region 2
KT361659	Amazona aestiva	ControlRegion2	Control Region	17183	18729	18853	+		.	T	INDEL				Control Region 2
KT361659	Amazona aestiva	ControlRegion2	Control Region	17183	18729	18853	+		.	C	INDEL				Control Region 2
KT361659	Amazona aestiva	ControlRegion2	Control Region	17183	18729	18853	+		.	G	INDEL				Control Region 2
KT361659	Amazona aestiva	ControlRegion2	Control Region	17183	18729	18853	+		.	G	INDEL				Control Region 2
KT361659	Amazona aestiva	ControlRegion2	Control Region	17183	18729	18853	+		.	T	INDEL				Control Region 2
KT361659	Amazona aestiva	ControlRegion2	Control Region	17183	18729	18853	+		.	C	INDEL				Control Region 2
KT361659	Amazona aestiva	ControlRegion2	Control Region	17183	18729	18853	+		.	A	INDEL				Control Region 2
KT361659	Amazona aestiva	ControlRegion2	Control Region	17183	18729	18853	+		.	T	INDEL				Control Region 2
KT361659	Amazona aestiva	ControlRegion2	Control Region	17183	18729	18853	+		.	T	INDEL				Control Region 2
KT361659	Amazona aestiva	ControlRegion2	Control Region	17183	18729	18853	+		.	G	INDEL				Control Region 2
KT361659	Amazona aestiva	ControlRegion2	Control Region	17183	18795	18853	+		T	C	transition				Control Region 2
KT361659	Amazona aestiva	ControlRegion2	Control Region	17183	18806	18853	+		A	G	transition				Control Region 2
KT361659	Amazona aestiva	ControlRegion2	Control Region	17183	18813	18853	+		C	.	INDEL				Control Region 2
KT361659	Amazona aestiva	ControlRegion2	Control Region	17183	18814	18853	+		A	.	INDEL				Control Region 2
KT361659	Amazona aestiva	ControlRegion2	Control Region	17183	18819	18853	+		T	A	transversion				Control Region 2
KT361659	Amazona aestiva	ControlRegion2	Control Region	17183	18824	18853	+		A	.	INDEL				Control Region 2
KT361659	Amazona aestiva	ControlRegion2	Control Region	17183	18826	18853	+		T	.	INDEL				Control Region 2
KT361659	Amazona aestiva	ControlRegion2	Control Region	17183	18827	18853	+		A	.	INDEL				Control Region 2
KT361659	Amazona aestiva	ControlRegion2	Control Region	17183	18829	18853	+		A	C	transversion				Control Region 2
KT361659	Amazona aestiva	ControlRegion2	Control Region	17183	18850	18853	+		T	C	transition				Control Region 2
